# Understanding AAV vector immunogenicity: from particle to patient

**DOI:** 10.7150/thno.89380

**Published:** 2024-01-20

**Authors:** Bijay P. Dhungel, Ian Winburn, Candida da Fonseca Pereira, Kui Huang, Amit Chhabra, John E. J. Rasko

**Affiliations:** 1 Gene and Stem Cell Therapy Program Centenary Institute, The University of Sydney, NSW, Australia.; 2 Faculty of Medicine and Health, The University of Sydney, NSW, Australia.; 3 Pfizer Inc, Walton on the Hill, Surrey, UK.; 4 Pfizer Australia, Sydney, NSW, Australia.; 5 Pfizer Inc, New York, NY, USA.; 6 Cell and Molecular Therapies, Royal Prince Alfred Hospital, Sydney, NSW, Australia.

**Keywords:** neutralizing antibodies, humoral immunity, seroprevalence, patient exclusion, redosing

## Abstract

Gene therapy holds promise for patients with inherited monogenic disorders, cancer, and rare genetic diseases. Naturally occurring adeno-associated virus (AAV) offers a well-suited vehicle for clinical gene transfer due to its lack of significant clinical pathogenicity and amenability to be engineered to deliver therapeutic transgenes in a variety of cell types for long-term sustained expression. AAV has been bioengineered to produce recombinant AAV (rAAV) vectors for many gene therapies that are approved or in late-stage development. However, ongoing challenges hamper wider use of rAAV vector-mediated therapies. These include immunity against rAAV vectors, limited transgene packaging capacity, sub-optimal tissue transduction, potential risks of insertional mutagenesis and vector shedding. This review focuses on aspects of immunity against rAAV, mediated by anti-AAV neutralizing antibodies (NAbs) arising after natural exposure to AAVs or after rAAV vector administration. We provide an in-depth analysis of factors determining AAV seroprevalence and examine clinical approaches to managing anti-AAV NAbs pre- and post-vector administration. Methodologies used to quantify anti-AAV NAb levels and strategies to overcome pre-existing AAV immunity are also discussed. The broad adoption of rAAV vector-mediated gene therapies will require wider clinical appreciation of their current limitations and further research to mitigate their impact.

## Introduction

Momentum has been increasing recently for gene therapy approvals worldwide following many years of setbacks 1, 2. A range of vectors (delivery vehicles, or carriers of therapeutic genes) have been explored for *in vivo* gene therapy designed to deliver genes of interest (transgenes) to target tissues. As adeno-associated virus (AAV) lacks significant clinical pathogenicity 3-5, it provides an ideal foundation for clinical gene transfer based on a recombinant AAV (rAAV) vector 6. rAAV can package and deliver transgenes to a wide variety of dividing and non-dividing cells and maintain stable, and potentially long-term, transgene expression (particularly in less rapidly- or non-dividing cells including the liver, retina and central nervous system) 3, 7. Further, AAV has a relatively low immunogenicity 3, 5 and optimized production protocols allowing for high-titer and high-purity manufacturing are available for clinical use 8, 9 (**Table 1**). Importantly, as the rAAV genome is predominantly maintained episomally in cells, complications related to insertional mutagenesis inherent to some other viral vectors used in clinical gene therapy are minimized 3, 4.

**Figure 1:**
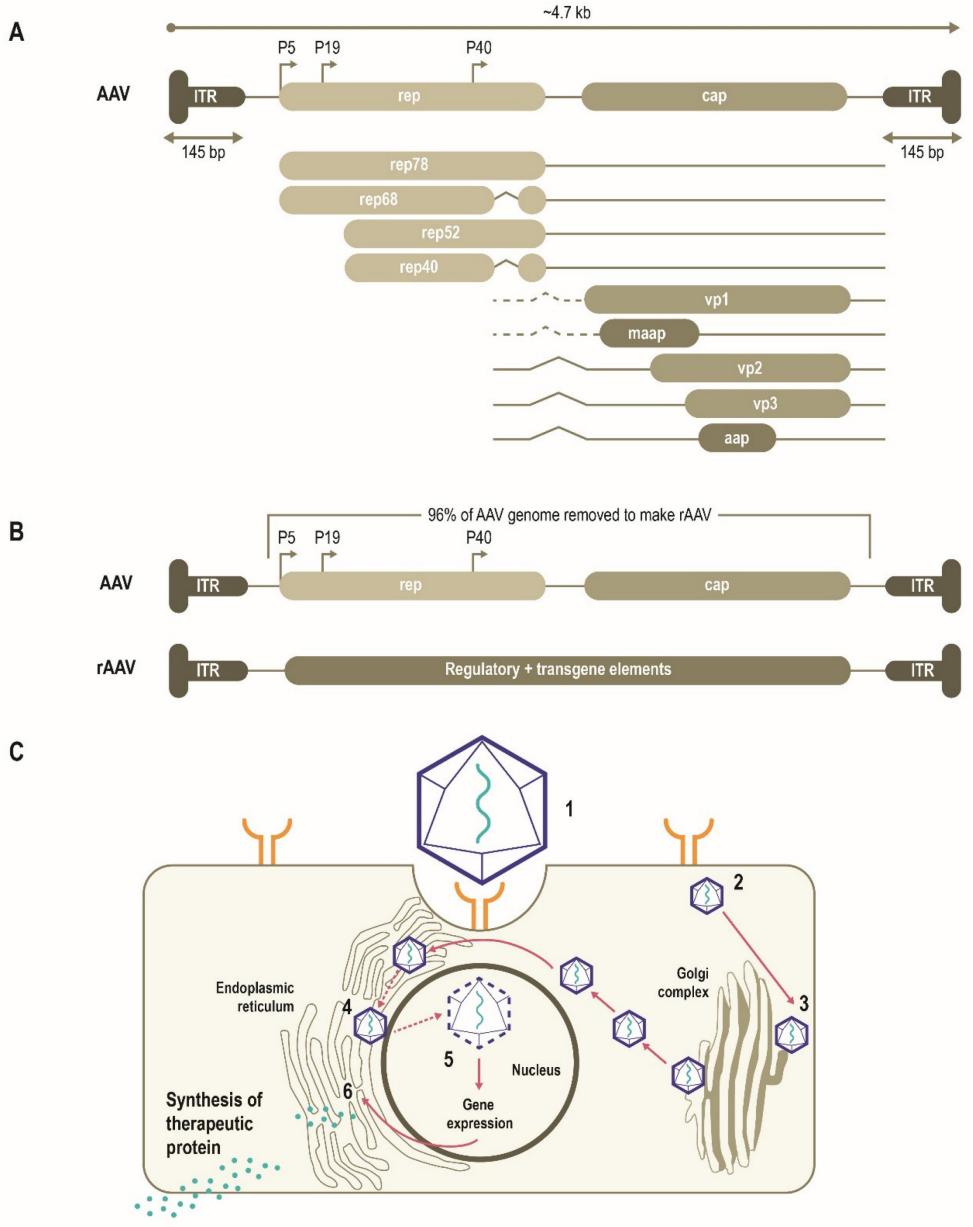
** Schematic representation of AAV genome, the AAV life cycle and the engineering of a generic rAAV expression cassette 10.** (**A**) AAVs consist of a single-stranded DNA genome of ~4.7 Kb enclosed by a capsid ~26 nm in diameter. The AAV genome contains three open reading frames flanked by two T-shaped inverted terminal repeats (ITRs). The *cap*, *rep, MAAP* and *AAP* of the AAV genome encode three capsid proteins, four replication proteins, the membrane-associated accessory protein (MAAP) that facilitates AAV egress and encapsulation and the assembly-activating protein (AAP) that facilitates capsid assembly in some serotypes. (**B**) In rAAV, the AAV genome is replaced by a transgene expression cassette, including a specialized promoter, enhancer, transgene of interest, and terminator, flanked with ITRs. (**C**) rAAV vectors transduce cells through (1) binding to cell surface receptors and/or co-receptors), followed by (2) internalization by endocytosis. AAV then (3) traffics through the endosomal and Golgi compartment and after endosomal escape, undergoes (4) nuclear transport and (5) uncoats releasing the genome, which is then (6) expressed. Relative size representations are not to scale.

AAVs consist of a single-stranded DNA (ssDNA) genome of ~4.7 Kb enclosed by a capsid ~26 nm in diameter (**Figure 1A**) 10, 11. The AAV genome is flanked by two T-shaped hairpin structures - inverted terminal repeats (ITRs) 10, 11. Overlapping genes with alternative splicing and multiple translation initiation sites allow for efficient use of the AAV genome and result in three capsid proteins (Cap), four replication proteins (Rep) as well as an assembly-activating protein that facilitates virion assembly in some serotypes 11. Together, these three genes mediate genome replication and packaging, capsid production, and integration 10, 11. More recently, an additional gene product coding for a membrane-associated accessory protein (MAAP) has been identified that facilitates AAV egress and encapsulation 12, 13.

**Figure 2:**
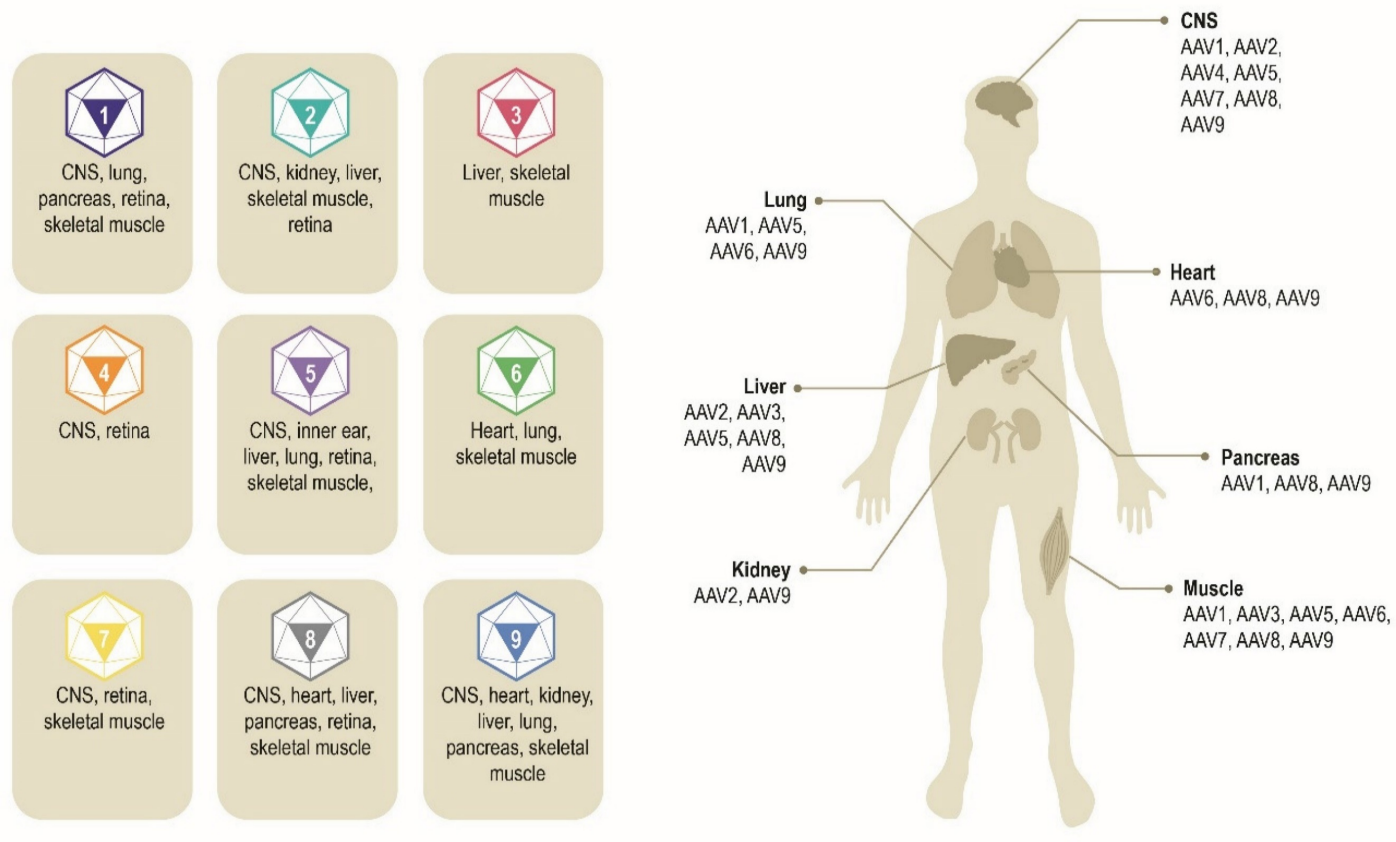
**Exemplar AAV serotypes and their preferential tissue tropism 6, 14, 195.** Differences in the capsid sequence and the presence of specific host cell receptor(s) determine cell/tissue tropism of exemplar AAV serotypes. AAV, adeno-associated virus. CNS, central nervous system.

Multiple AAV serotypes exist with the same overall genome structure 4, 10, 14, 15 and over 80% homology in nucleotide sequence 16. The difference between serotypes is primarily within the amino acid sequence of their capsid proteins 14, 17. It is the differences in the capsid sequence and the presence of receptors or co-receptors on the host cell that determine cell/tissue tropism of a particular AAV serotype (**Figure 2**) 10, 14, 18, 19. AAV2 is the most common serotype and is close in amino acid sequence similarity to all AAV serotypes except AAV4 and AAV5 17, 20. A comparison of phylogenies from human and non-human primate AAV serotypes revealed that human AAV4 and AAV5 serotypes are the most divergent, while the other human serotypes (AAV1, AAV6, AAV2, AAV3, and AAV9), unique non-human primate serotypes (AAV7), or serotypes found in both human and non-human primates (AAV8) clustered in groups 17, 21. AAV1, AAV2, and AAV3 are closely related to each other, with 83% to 93% homology at the amino acid level 20. AAV4 and AAV5 are more distantly related to AAV1, AAV2, and AAV3 based on the amino acid sequence of viral proteins VP1/VP3 ranging from 51% to 63% sequence identity for AAV4 20 and 51% to 59% for AAV5 20, 22, 23. AAV6 is a hybrid of AAV1 and AAV2, with 99% homology to AAV1 16, 24.

AAV has been bioengineered to produce rAAV vectors to target diverse monogenic disorders, and many therapies are either approved or in late-stage development (**Table 2**). In a rAAV vector, the AAV genome is replaced by a transgene expression cassette that includes a specialized promoter, transgene of interest, and transcriptional terminator, flanked with ITRs (**Figure 1B**) 11, 25. In addition to accommodating a transgene expression cassette, this replacement of viral coding sequences contributes to lower immunogenicity and cytotoxicity (**Table 1**) 11. The tissue tropism of the resultant rAAV is largely determined by the capsid used with a range of natural or engineered capsids available to target different tissues 11, 14, 25. The selectivity and efficiency of transduction can be further enhanced by optimizing the transgene expression cassette through improved codon usage, CpG-depletion or with the inclusion of tissue- or cell-selective regulatory elements 11, 26, 27. AAV enters cells through receptor-mediated endocytosis (**Figure 1B**) 10, 11, 28. AAV then traffics through the endosomal and Golgi compartments and, after endosomal escape, undergoes nuclear transport and uncoating 10, 11. The single-stranded AAV genome converts into a double-stranded genome through the activity of cellular DNA polymerase 10, 11. AAV requires a helper virus (e.g., adenovirus, herpes simplex) to facilitate gene expression and viral replication 10, 11. In the presence of a helper virus, AAV undergoes productive infection characterized by genome replication, viral gene expression, and virion production 25. Without helper virus, the AAV can establish latency by undergoing integration into the adeno-associated virus integration site 1 (AAVS1) on chromosome 19, and also at other chromosome locations mediated by the Rep protein 25, 29. Currently, no confirmed cases of rAAV-mediated genotoxic events have been reported in humans to date. However, due to some reports of insertional mutagenesis in murine studies, research on genomic integration of rAAV is of active interest 29, 30.

## Challenges for rAAV gene delivery

### The Humoral Immune Response

**Figure 3:**
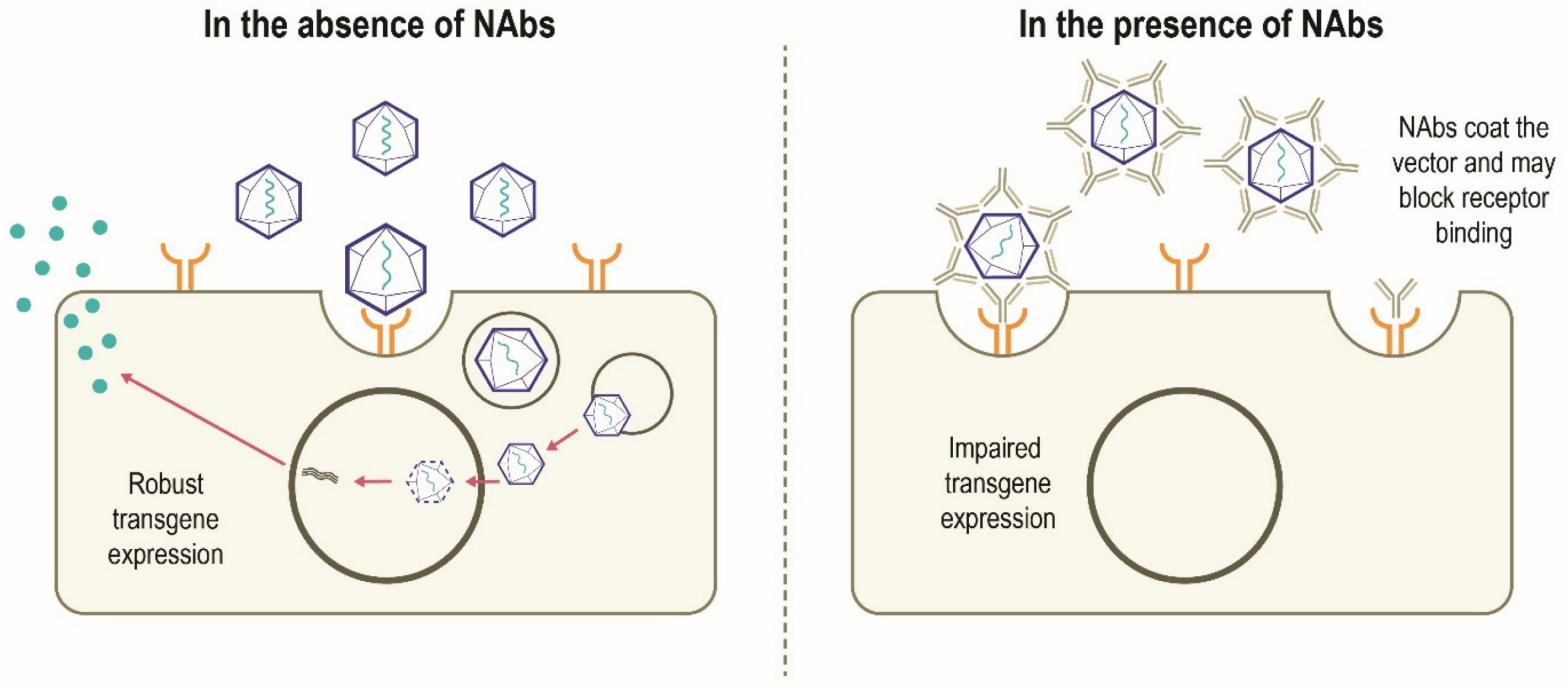
**Canonical mechanism of action of NAbs 37, 83.** In the absence of pre-existing NAbs, robust transgene expression is possible. In the presence of NAbs, developed following previous exposure to AAV, NAbs bind to the AAV vector and may prevent binding to the target cell receptor which can block vector transduction and transgene expression. The NAb may also inhibit the interaction of viral envelope protein and cell-surface receptors after the vector has bound to the cell. Other mechanisms by which NAbs impair transgene expression not shown include NAb-coated vector binds to the cellular receptor and is internalized and subsequently destroyed intracellularly; the NAbs bind to the cell receptor and block the rAAV binding and subsequent internalization. NAb, neutralizing antibody; rAAV, recombinant adeno-associated virus.

Although AAV possesses several features to make it an attractive gene therapy vector, there are challenges to be overcome in the development of widespread successful AAV-mediated therapies (**Table 1**). Despite having a low immunogenicity profile 3, 5, rAAV vectors can stimulate host antiviral immune responses directed against the capsid and/or the encoded transgene product, particularly when delivered systemically or at higher vector doses 14, 31. Anti-AAV neutralizing antibodies (NAbs) can develop following exposure to naturally occurring AAVs, with increased population seropositivity correlating with age 32-34. Passive transfer of maternal anti-AAV antibodies also occurs 33, 35. A high degree of homology between AAV capsids allows these NAbs to cross-react with different capsids, including those used for gene therapy 14, 36. A humoral immune response against the capsid is triggered in patients who have received gene therapy, which subsequently results in the neutralization of cross-reactive rAAVs administered 6, 14. In this scenario, NAbs may coat the rAAV vector and 'block' its binding to the receptor(s) on the target cell and thus prevents attachment and cell entry. Alternatively, NAbs may inhibit the interaction of viral envelope protein and cell-surface receptors after the vector has bound to the cell or otherwise inhibit transduction, thereby limiting the efficacy of gene therapy. It is also possible that the NAb-coated vector binds to the cellular receptor and is internalized but productive transduction is inhibited. Alternatively, the NAb itself may bind to the cell receptor and block the rAAV binding and internalization (**Figure 3**) 37. However, it has been suggested that some AAV serotypes may be less sensitive to antibody neutralization, with partial resistance to neutralization observed in recent trials of hemophilia B gene therapy using AAV5 38-40. NAbs may also impact vector biodistribution, directing the AAV vector away from target cells towards secondary lymphoid organs 41. The level of anti-AAV NAbs depends on diverse patient- and therapy-related factors, including patient demographics, disease state, capsid type, transgene, and dose 14. Approaches to minimize the effect of humoral immunity against AAV are being explored, but significant hurdles remain.

In addition to pre-existing anti-AAV NAbs that impact the initial transduction of rAAV, host cellular immune responses may reduce the long-term durability of the transgene expression 42, 43. T-cell-mediated immunity post-cellular entry may include recognition of the capsid by toll-like receptor (TLR) 2 at the cell surface and stimulation of TLR9-mediated innate immunity. The degradation and presentation of Cap on major histocompatibility complex (MHC) class I molecules may result in the clearance of transduced cells by capsid-specific cytotoxic CD8^+^ T cells 44 and presentation of capsid proteins on MHC class II molecules 11, 42. In turn, the MHC class II complex may be recognized by CD4+ T lymphocytes, resulting in the secretion of interleukins and stimulation of B lymphocytes 42. This can lead to an anti-capsid humoral response 28, 42, thereby reducing long-term persistence of the therapy or preventing successful vector re-administration.

#### Seroprevalence of NAbs

Considering the prevalence of AAV infection, cross-reactivity of NAbs, and potential for NAb-mediated inhibition of rAAV-mediated gene therapy, an understanding of the seroprevalence of AAV NAbs is important. Seroprevalence has been studied in healthy individuals as well as in patients potentially amenable to gene therapy. Seroprevalence profiles for AAV serotypes 1 to 9 and bioengineered capsids in humans vary by geography, race, and age as well as assay methodologies and their sensitivities (**Table 3**). It is important to note that the seroprevalence studies discussed here focus largely on AAV. Therefore, caution with respect to the direct applicability of these data to specific rAAVs used in gene therapy trials is warranted.

##### Geographical differences in seroprevalence

One large population-based study of healthy volunteers in 10 countries across four regions 45 showed anti-AAV2 NAbs were the most prevalent antibodies in all regions (30%-60%), followed by anti-AAV1, anti-AAV7, and anti-AAV8 NAbs 45. The prevalence of anti-AAV1, anti-AAV7, and anti-AAV8 NAbs was lower in the United States compared with other countries 45. In another study of healthy volunteers in the United States and Europe, the prevalence of anti-AAV2 and anti-AAV8 NAbs was high although some regional variations were noted 46. Another observational, retrospective study of participants from 10 countries (Australia, Canada, France, Germany, Italy, Japan, South Korea, Spain, United Kingdom, and United States) who were enrolled in non-gene therapy trials showed that anti-AAV1 NAbs were the most prevalent (74.9%), followed by AAV6 (70.1%) and AAV5 (63.9%) 47. However, prevalence varied by dilution used in the assay, with AAV5 having the lowest seroprevalence 47. Overall, the prevalence of all AAV serotypes studied was highest in South Korea and lowest in Japan, Australia, and the United States 47.

Considerable geographic variability across serotypes has also been observed in individuals with hemophilia A, particularly for anti-AAV5 NAbs. In one study (N=546) across nine countries, AAV5 consistently exhibited the lowest seroprevalence of all serotypes 32. The global weighted average (factoring in the country specific prevalence of hemophilia A) of AAV5 seroprevalence was 29.7%, with seropositivity rates ranging from 5.9% in the United Kingdom to 51.8% in South Africa. Another study showed the prevalence of anti-AAV5 NAbs ranged from 21% in the United States to 47% in Russia 48.

##### Racial differences in seroprevalence

In a study in the United States of healthy donors who self-identified as belonging to a race, prevalence of NAbs to most AAV serotypes was higher among Black and Hispanic donors compared with those who self-reported as White 49. The study of non-gene therapy trial participants from 10 countries showed a higher seroprevalence in Asians compared with non-Asian participants 47.

##### Age differences in seroprevalence

Increases in the seroprevalence of anti-AAV NAbs have been observed with age both in healthy individuals 50, 51 and in a number of disease states 32, 34, 47, 51. One study examining anti-AAV NAb seroprevalence from birth to adolescence in the United States (N=752) reported that anti-AAV2 and anti-AAV8 NAbs were higher in neonates (59% and 36%, respectively) but declined substantially in infants aged 7 to 11 months and then increased again in children and adolescents aged 3 to 18 years 33. Interestingly, in a smaller study of patients with mucopolysaccharidosis (MPS) III and healthy individuals, the seroprevalence differed between cohorts. NAbs against all AAV serotypes were higher in healthy children aged 8 to 15 years than in those aged 2 to 7 years, whereas in children with MPS III the opposite was observed, with seroprevalence peaking before 8 years of age 34.

It is important to note that not all studies have reported an association between age and seroprevalence. For example, no age-related differences in AAV2, AAV3, AAV8, or AAVLK03 were noted in a smaller study of healthy Chinese participants (N=100) ranging in age from ≤35, 36 to 50, and ≥51 years 52.

##### Gender differences in seroprevalence

Some studies have proposed that the prevalence of anti-AAV NAbs can be influenced by gender. The prevalence of anti-AAV1, anti-AAV5, and anti-AAV8 NAbs was significantly higher in healthy Chinese women than male counterparts 53. Similarly, another study found higher level of anti-AAV9 NAbs in adult females 47. However, not all studies have reported significant gender differences in anti-AAV1 NAbs 54. Intraracial differences have also been observed between genders, with White women and Hispanic men more seropositive compared with their gender counterparts 49.

##### Differences in seroprevalence according to disease state

The seroprevalence of NAbs against AAV also varies across disease states relative to healthy populations (**Table 4**). Evidence suggests that seropositivity in patients may be affected by the nature of the disease and/or treatment received. There are inconsistencies in exact rates across studies that could be explained by differences in study design and implementation (i.e., assay type: see section 'Preparing for rAAV gene therapy: screening for anti-AAV NAbs' 55), geographic location, or other factors such as age, but seroprevalence trends are similar 32, 45, 47. For example, anti-AAV2 NAbs were the most prevalent antibodies across disease states, including hemophilia 56, cystic fibrosis 57, rheumatoid arthritis 58, and primary Sjögren syndrome 59. Furthermore, the prevalence of anti-AAV NAbs increased with age in patients with hemophilia A and cystic fibrosis 56, 57, 60.

There are interesting reports regarding anti-AAV NAbs among individuals who receive plasma-derived blood products. One such study noted an increase of anti-AAV8 NAbs in patients with hemophilia A exposed to plasma products, as well as an increase in anti-AAV5 and anti-AAV8 NAbs in patients exposed to hepatitis C 60. Although the authors noted a possibility of transfusion-transmitted infection, they acknowledged further investigation was required to establish the cause 60. This was explored further using a highly sensitive assay to detect AAV gene sequences in a range of commercially available plasma and recombinant factor VIII (FVIII) and factor IX (FIX) products 61. Results indicated a presence of AAV and other viral serotypes in some of the plasma-derived blood products. In contrast, another study did not find an association between anti-AAV6 NAbs in patients with hemophilia B and exposure to contaminated plasma derivatives 62. This apparent discrepancy could be explained by the AAV serotype under study. Neutralizing activity against multiple AAV serotypes is also possible, either through cross-reactivity of NAbs or the co-occurrence of NAbs as a result of multiple or co-infections within the same individual. This creates profound implications when attempting to implement alternate serotypes or engineered capsids in clinical practice 34, 46, 57.

An issue raised more recently relates to the development of anti-AAV NAbs in recipients of rAAV-based vaccines, including those against SARS COV-2 63. With the high frequency of cross-reactivity among different AAV serotypes, such vaccines could render recipients with genetic disorders ineligible for future rAAV-mediated gene therapy. This is a topic requiring further discussion and research.

#### Pre-existing NAb titer and post administration increases in NAbs

There are conflicting data regarding the relationship between titers of pre-existing anti-AAV NAbs and the efficacy of vector transduction. The presence of anti-AAV NAbs increases dramatically after administration of rAAV vectors (**Table 5**), which has implications for redosing 64. In the first hemophilia B gene therapy clinical trial that used rAAV2 expressing human FIX (AAV-hFIX), there was a greater than 10,000-fold increase in anti-AAV NAb titers after vector administration. This was true for participants with or without detectable anti-AAV NAbs pre-treatment 65-67. Therapeutic levels of FIX were achieved but reduced approximately 8 weeks post-infusion, correlating with the development of capsid-specific T cells 65, 66.

In most gene therapy trials utilizing rAAV, pre-existing anti-AAV NAbs are an exclusion criterion for patient enrollment 34. For example, patients with pre-existing anti-AAV9 NAbs above a threshold were excluded from a phase 1 spinal muscular atrophy clinical trial 68 as were patients with anti-AAV5 NAbs from a phase 3 clinical trial for hemophilia A 69. However, some findings have challenged exclusion based on this criterion 38, 39. For instance, pre-existing anti-AAV5 NAbs had no effect on the efficacy of AAV5-based gene therapy expressing hFIX in a phase 1/2 hemophilia B gene therapy trial (NCT02396342) 70, even at high titers 39. Similarly, there was no correlation between pre-existing anti-AAV5 NAbs and AAV5-mediated delivery of hFIX up to 18 months in a phase 3 hemophilia B trial (NCT03569891) 38. Given that the observed increase in mean FIX activity was restored to near normal range at 18 months 38, these trials imply broad eligibility for AAV5-based therapies 38, 39, 71. The NCT03569891 trial excluded patients with pre-existing anti-AAV5 NAbs based on a green fluorescent protein (GFP)-based reporter assay 39, 70. In a follow-up study using a more sensitive luciferase-based assay, immunoglobulin (Ig)G and IgM antibodies against AAV5 were detected 39, 70. These observations highlight the importance of the assay being used to examine NAb levels.

Apart from NAbs 6, 14, memory B cells and T cells are also generated due to natural AAV infection that can be re-activated on subsequent exposure to rAAV vectors 42, 73. An investigation of immune responses to natural AAV1 infection by screening human peripheral blood mononuclear cells and sera from healthy donors showed no correlation between AAV1-specific T cells and anti-AAV1 NAb responses 74. T-cell response composed mostly of effector memory CD8+ cell subsets. This suggests that patient screening should include testing for pre-existing AAV-specific cellular responses in addition to anti-AAV NAbs 74.

There is a need to standardize the quantitative relationship between neutralizing or total antibody titers and their impact on transduction for individual AAV serotypes 71. It is important that validated assays to determine NAb titers are made available as gene therapy products are commercialized. The absence of validated assays to accompany regulatory approvals of some gene therapy products leaves clinicians with a challenging task of evaluating results from non-validated NAb assay kits.

### Systemic Inflammatory Responses

Administration of gene therapy to patients with pre-existing NAbs can induce systemic inflammatory responses due to immune complex formation, enhanced vector uptake into antigen presenting cells (APCs), and complement activation, especially at higher titers. One study demonstrated that titers of NAb ≥1:100 significantly increased the innate immune response to AAV vectors with increased pro-inflammatory cytokine/chemokine secretion, vector uptake by APCs, and complement activation 75. As complement is an important modulator of the anti-AAV immune response, inhibiting the complement pathway could help mitigate anti-AAV immune responses 76.

## Preparing for rAAV gene therapy

### Screening for Anti-AAV NAbs

**Figure 4:**
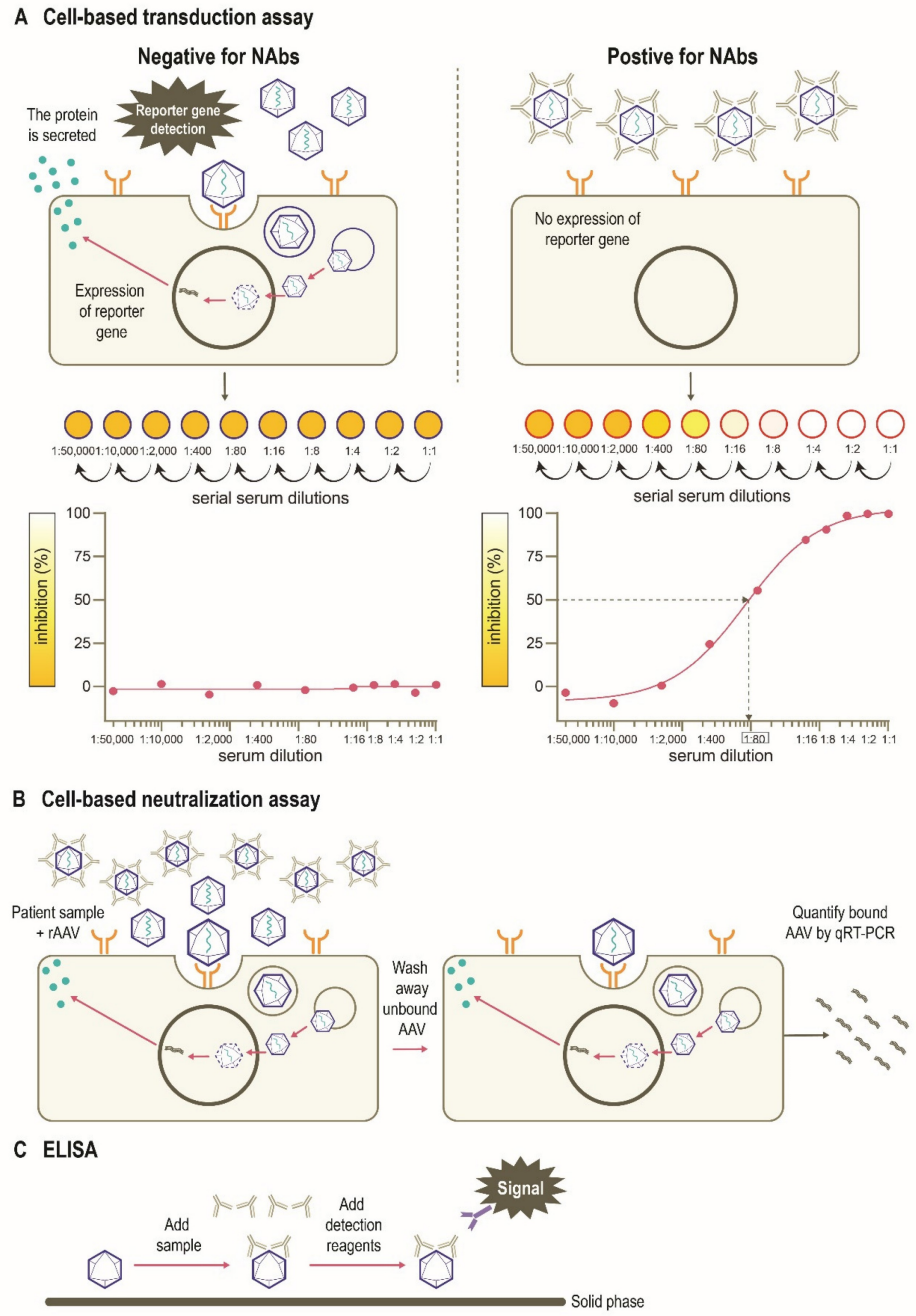
** Assays used to detect immunity to AAVs.** (**A**) An *in vitro* cell-based transduction inhibition assay, using a “reporter” gene such as GFP, β-galactosidase, or luciferase, which provides convenient and sensitive detection of transduction 71, 77, 78. The degree of inhibition of reporter gene expression is plotted against the dilutions of the serum sample. The anti-AAV NAb titer is defined as the highest serum dilution that inhibits vector transduction by a specified amount (eg, ≥50%) in comparison with the negative control sample. (**B**) An *in vitro* cell-based neutralization assay measures the binding of rAAV to target cells. Increased levels of NAbs are associated with a proportionate decrease in rAAV cell binding 71, 80, 84. (**C**) ELISAs can be used to measure antibody binding to the AAV capsid or other serotype-specific proteins or peptides. This method includes coating of the assay plate with AAV capsids (full or empty) or peptides, the addition of patient sample, and finally detection of signal 77, 78, 83. AAV, adeno-associated virus; ELISA, enzyme-linked immunosorbent assay; GFP, green fluorescent protein; NAb, neutralizing antibody; qRT-PCR, quantitative reverse transcription polymerase chain reaction; rAAV, recombinant adeno-associated virus; IC50, half maximal inhibitory concentration.

There are several assays to quantify neutralizing and total (non-neutralizing plus neutralizing) anti-AAV antibodies in humans, including *in vitro* cell-based assays and variants of the enzyme-linked immunosorbent assay (ELISA) (**Figure 4**) 77. The protocols and reagents are not standardized and are usually adapted to accommodate the AAV serotype and/or transgene under investigation 78. The most widely used *in vitro* cell-based assay is the transduction inhibition assay, which involves the measurement of transduction levels using a “reporter” gene (**Figure 4A**). Instead of the therapeutic transgene, a rAAV vector contains a reporter gene such as GFP, β-galactosidase, or luciferase, which provides a convenient and sensitive measure of transduction at wide dynamic ranges. The ability of NAbs to inhibit transduction of the specific AAV vector is then assessed by measuring the activity of the reporter protein (**Figure 4B**). The anti-AAV NAb titer is defined as the highest dilution that inhibits transgene expression by a specified amount (e.g., ≥50% [ID_50_]) 71, 77-79. An advantage of this method is that it uses the same AAV capsid engineered into the gene therapy vector, recapitulating the clinical setting 71. However, the output (e.g., 50% inhibition of transduction) may not correlate to a biologically relevant patient response 71. As the assay measures reduction in transduction, factors other than NAbs may be involved. For example, not all AAV serotypes transduce efficiently *in vitro*, and the sensitivity of the assay may depend on the number of AAV vector genomes or multiplicity of infection (MOI), as well as the cell line and reporter system used 71, 80. Indeed, the luciferase reporter system is likely to be more sensitive than a GFP-based reporter 39, 72, 81. Additionally, the purity of the vector preparation influences NAb titer with the presence of monomeric or oligomeric capsid proteins, “empty” vectors, or vectors containing truncated genomes or genomic DNA potentially affecting the outcome of this assay 79. Inhibition by other factors in the serum that reduce transduction such as human serum galectin 3 binding protein (G3BP) 82 can also impact the assay sensitivity. Furthermore, these assays are often proprietary, and therefore standardization and broad application remains challenging. Thus, there are pressing opportunities to harmonize such assays within and between jurisdictions as clinical gene therapies become widespread.

An alternative *in vitro* cell-based assay is the neutralization assay, which measures the binding of AAV vectors to target cells (**Figure 4C**) 71, 83. Since anti-AAV NAbs can interfere with the binding of AAV to target cells, increased levels of NAbs in culture are associated with a measurable decrease in rAAV cell binding. The ability of the NAb to inhibit AAV binding is often assessed by measuring the activity of a reporter protein 71, 83. As many cell-based assays are time-consuming 71, newer methods have been developed to determine NAb titers that use real-time quantitative reverse transcription polymerase chain reaction (qRT-PCR) methodology as opposed to reporter genes and can be applied to all serotypes in a fast, efficient, and cost-effective manner (**Figure 4C**) 80. Other rapid cell-based assays have also been developed to provide alternative methods for *in vivo* determination of NAb titers 84. However, the neutralization assay does not directly inform overall gene transfer efficiency or transduction as steps subsequent to cell binding are not evaluated 83.

Total antibody assays, such as ELISAs, can be used to measure antibody binding to the whole AAV capsid or capsid proteins 77, 78, 83. These typically involve coating of the assay plate with AAV capsids (full or empty) or peptides, the addition of patient sample, and finally, detection of signal (**Figure 4D**). Peptide-based ELISAs can be more sensitive, specific and consistent than capsid ELISAs 85. The peptide-based ELISA method has the advantage of simplicity compared with cell culture protocols, although neutralizing activity is not measured directly and levels of total anti-AAV antibodies are captured 77, 80. The total antibody assay measures both neutralizing as well as non-neutralizing antibodies against AAV. Evidence suggests a correlation between levels of total anti-AAV antibody and NAbs 46, 86. There have been studies suggesting that non-neutralizing antibodies may enhance the transduction rates of some serotypes including AAV8 41 although mechanisms behind this phenomenon are yet to determined. Thus, the use of total antibody assay to determine a patient's eligibility for AAV gene therapy needs to be studied further 79.

Currently, beyond the lack of standardized assays, variations exist in cut-off values for NAb positivity between studies 31, 77, 83. This makes comparison of NAb titers across studies difficult, particularly when the details of the assays used are lacking. Clinical trials conducted in patients with hemophilia exemplify this issue with differences in the cut-offs and types of assays used. For instance, in a rAAV5-based vector expressing FVIII in men with severe hemophilia A (NCT02576795) 87, exclusion was based on an *in vitro* transduction inhibition assay with a cut-off of 44.9% transduction and a total anti-AAV5 antibody assay cut-off of signal dilution ≥39.7%. The transduction inhibition assay employed the HEK293T/17 cell line, a rAAV5 vector expressing a luciferase reporter and a MOI of 25,000 vector genomes (vg)/cell 87. A separate trial using a rAAV5-based vector expressing wild-type human FIX in adults with hemophilia B (NCT02396342) 70, exclusion was based on a 29% inhibition of transduction. The assay employed a rAAV5 vector expressing a GFP reporter, but no details of the MOI were provided. In another phase 2b trial using a rAAV5-based vector (NCT03489291) 88, patients with detectable anti-AAV5 NAbs titers were included. The NAb level was determined using the HEK293T cell line, a rAAV5 vector expressing a luciferase reporter and a MOI of 378.4 vg/cell 39, 88.

A standardized approach to reporting the number of AAV vector particles neutralized per unit volume (e.g., μL) of serum or plasma will be essential as stakeholders, including clinicians, scientists, industry, consumers and regulators, seek to compare trial results 79.

#### NAb screening to determine eligibility for AAV-based gene therapy

The titer of NAbs is routinely assessed prior to the administration of AAV gene therapies and is important given that even low titers can potentially to inhibit vector transduction especially after systemic administration 89. Guidelines have proposed the development of appropriate assays to measure immune responses to gene therapy products early in the development process to monitor outcomes and inform treatment decisions 90. However, there is no clear, consistent relationship between pre-existing anti-AAV NAb titers and clinical vector transduction levels 38, 39, 65, 66, 91. In a recent meta-analysis of clinical AAV usage, it was determined that 45% of trials excluded subjects with pre-existing anti-AAV Nabs. This varies between therapeutic areas, with the proportions ranging from <10% for eye disorders to ~90% for blood disorders 6.

A recent phase 1/2 clinical study of an AAV5-mediated gene therapy for severe hemophilia A excluded patients with pre-existing anti-AAV5 NAbs 92. Immunogenicity data up to 3 years suggested the predominant immune response elicited by vector infusion was largely limited to the development of an anti-AAV5 antibody response with cross-reactivity to other serotypes, including AAV2, AAV6, AAV8, and AAVrh10 92. Patients with pre-existing anti-AAV5 NAbs were also excluded from another phase 3 study (NCT03370913) of hemophilia A that successfully reduced bleeding and the need for FVIII concentrates in participants 69. In contrast, another study is currently recruiting patients with pre-existing anti-AAV5 NAbs 93, which will help inform whether NAbs should be an exclusion criterion especially for AAV5-based gene therapy clinical trials.

Other recent studies demonstrate that NAbs may not always impair the efficacy of gene delivery, as in the phase 1/2 trial (NCT02396342) discussed earlier 39, 70. Indeed, the highest post-infusion FIX activity in this trial was observed in a participant with the highest pre-existing anti-AAV5 NAb titer of 1:340 39, suggesting efficacy is not always related to anti-AAV NAb titer. In a phase 2b trial (NCT03489291) of a different AAV5-based gene therapy for hemophilia B and with pre-existing anti-AAV5 NAbs; all participants met the primary efficacy endpoint and had sustained FIX production and protection from spontaneous bleeding at the initial 26-week follow-up and after the 2-year follow-up period 88, 94. Accordingly, pre-existing anti-AAV5 NAbs were not used as an exclusion criterion in the subsequent phase 3 trial (NCT03569891). The 18- and 24-month follow-up data demonstrated no correlation between therapeutic benefit and pre-existing anti-AAV5 NAbs 38. Of note, one of the 54 participants enrolled had a high anti-AAV5 NAb titer of 3212 prior to vector dosing and did not respond to treatment 38.

These findings suggest clinically meaningful outcomes in patients with NAb titers at levels found in the general population may be achievable and may therefore enable a larger proportion of patients to potentially benefit from AAV-based gene therapies.

The full extent of the influence of vector dose on outcomes and clinical impact in the long-term is unclear and requires further investigation. It is also possible that AAV5 serotype is unique and less sensitive to NAbs 40. Given the impact of pre-existing NAbs on outcomes, reliable *in vitro* assays to detect anti-AAV NAbs are required, not only for patient selection, but also to inform the clinical management of patients before, during and after treatment.

### Strategies to Mitigate Anti-AAV NAbs

#### Strategies to mitigate pre-existing anti-AAV NAbs

With the majority of the global population exposed to AAV 6 and who potentially may have high levels of pre-existing NAbs against specific AAV serotypes (**Table 3**), it is important to develop strategies to mitigate the risks and to potentially expand the gene therapy-eligible patient population (**Table 6**) 5, 79. Administering a high vector dose, particularly in the presence of low titer NAbs, may be an option to overcome the effects of NAbs 66 . However, both clinical and pre-clinical studies have demonstrated dorsal root ganglia toxicity after administration of high doses of AAV vectors through the cerebrospinal fluid (although no clinical signs were observed) 95, 96.

Modifying the mode or route of administration may be another option to reduce the humoral immune response to AAV-mediated gene therapy. A study conducted in macaque monkeys suggested portal vein-directed delivery of AAV8-based vectors with saline flushing is efficacious to minimize the inhibitory effect of pre-existing anti-AAV8 NAbs 97. Similarly, intramuscular injection of a rAAV2-based gene therapy for hemophilia B was shown to result in successful gene transfer in patients with pre-existing anti-AAV NAbs, suggesting this method is less susceptible to the neutralizing activity of the NAbs 65. It should be noted that despite successful gene transfer, circulating FIX were below therapeutic levels in this trial and capsid-specific T-cell responses can be triggered after intramuscular injection of rAAV vectors 98.

Identifying AAV capsids with altered epitopes that might be able to avoid the neutralizing activity of pre-existing NAbs offers another strategy. Alternatively, an AAV serotype with a lower seroprevalence may be used, as more than 100 naturally occurring AAVs have been identified. For instance, a comparison of NAb levels between AAV2, AAV4, AAV5, AAV12, and BAAV in serum of 39 patients with Sjögren disease and 38 healthy donors discovered lowest seroprevalence for AAV12 in both groups 59. AAV vectors can also be engineered by directed evolution or rational design by mutagenesis of AAV capsids - this may lead to capsids with distinct tissue tropism, immunogenicity, and/or susceptibility to NAbs 5, 99-103. However, artificial engineering of capsids for human gene therapy is associated with several challenges. Firstly, designing a capsid with all desired properties (tropism, immune evasion, packaging ability, and safety) is inefficient. This process may alter natural biology, decrease packaging ability, effect large-scale production, or even increase the antigenic load 104. An assessment of all these aspects for each new capsid is required in pre-clinical as well as clinical settings. Nonetheless, several reports suggest that AAV capsid modification can lead to NAb evading vectors.

Additional non-genetic, chemical modifications of the AAV capsid may avoid the humoral immune response and neutralization by NAbs. For example, conjugating the AAV surface with polyethylene glycol chains (PEGylation) may protect AAV vectors from NAbs 105. The inclusion of antibody decoys such as empty capsids in the final vector formulation may overcome the inhibitory effect of NAbs 106. However, empty capsids may also contribute to the overall number of capsid antigens presented onto MHC class I and may also have an impact on the immunogenicity of AAV vectors 28, 107.

Immunosuppressants, given during or shortly after gene transfer, are often used to prevent the rejection of rAAV-transduced cells 5, 108 . However, despite their demonstrated effectiveness at dampening anti-AAV T-cell response, circumventing pre-existing NAbs using this method is challenging 5. Plasmapheresis, with selective *ex vivo* removal of AAV-specific NAbs from an individual's blood using filtration- or centrifugation-based techniques may help manage seropositive patients and enable successful AAV transduction 5, 109. Similar results might be possible using pre-treatment with immunoglobulin degrading enzymes, such as imlifidase, that can cleave IgG antibodies in seropositive patients to reduce pre-existing NAb levels 110-112. Although these strategies have modestly decreased titers, they have not eliminated the presence of NAbs. It remains to be determined whether one or combination of some or all of the above-mentioned strategies will be most beneficial to mitigate the impacts of pre-existing NAbs.

#### Strategies to mitigate post-administration humoral immunity against rAAV vectors

As levels of anti-AAV NAbs can increase dramatically after administration of rAAV vectors, preventing re-administration of the same vector 64, it is important to develop strategies to mitigate humoral immunity to rAAV vectors post-administration (**Table 6**). Minimizing vector dosage is one such strategy, as rAAV vector immunogenicity appears to be dose dependent 113, 114. It may be possible to manage expression of the transgene with low doses of vector together with broad-acting immunosuppressants such as corticosteroids 5, 114, 115. These have been employed in therapies approved for inherited retinal dystrophy (1·5 × 10^11^ vg) 116 and in development for Crigler-Najjar syndrome (2 × 10^12^ vg/kg to 5 × 10^12^ vg/kg doses) 117. Downregulating CD4+ helper T cells can be used to reduce anti-AAV NAb levels indirectly due to their ability to regulate the function of B cells and the antibody producing progeny plasma cell 5. For instance, inhibiting B-cell activation has shown promise in several pre-clinical ocular models 5. Reducing off-target expression of the transgene with tissue-specific promotors can also limit immune responses directed towards the transgene 118. Effective strategies to overcome pre-existing NAbs and prevent the induction of humoral immunity against AAV gene therapies are likely to include a combination of approaches, particularly if the patient has a high pre-existing NAb titer.

### Considerations for Pediatric Patients

There are specific considerations for pediatric patients receiving AAV-based gene therapy. When considering the challenge of pre-existing anti-AAV NAbs, seroprevalence is the lowest in patients under the age of 3 years and progressively increases into adulthood 33, 56 This may be an optimal therapeutic window, when the risk of anti-AAV NAbs is at its lowest and the proportion of eligible patients for gene therapy is at its highest 28. However, when AAV vectors are delivered to pediatric patients, transgene expression may dilute with liver growth and increased blood volume 119, 120. A single administration of an AAV-based vector may be sufficient to achieve a lifelong benefit for diseases such as hemophilia, with a relatively low therapeutic threshold 120, where even a low expression of the transgene may convert the disease phenotype from severe to mild 113. As liver cells proliferate from birth to teenage years, episomal rAAV is expected to dilute, possibly leading to a loss of therapeutic benefit 121. For diseases requiring robust transgene expression or treatment in childhood, vector re-administration or a methodology that uses vector genome integration or genome editing may be required 28.

## Conclusions

There is compelling evidence that rAAV has become an established vector for human gene therapies. Although a number of clinical trials have shown the efficacy and safety of rAAV-mediated gene transfer, NAbs (pre-existing or induced post-therapy) remain a significant challenge. Seroprevalence profiles for AAV serotypes in humans vary by region, geography, race, and age. As NAb levels are an important patient exclusion criteria in clinical trials 53, it is important for healthcare professionals preparing for gene therapy to understand the challenges associated with NAbs and their impact on eligibility for clinical trials in AAV-based gene therapy, as well as strategies to mitigate their impact. Clarifying the relationship between NAb titer and its impact on transduction for individual AAV serotypes is required. This is especially the case for capsids like AAV5 that seem to behave differently in the presence of NAbs compared with other AAV serotypes. Clinical gene therapies require a consistent approach when reporting the number of AAV vector particles neutralized per unit volume of serum or plasma. Comprehensive and combinatorial strategies will likely be required to mitigate AAV NAbs to overcome pre-existing and post-therapy humoral immune responses. Gene therapy trials are often small, single-arm, short-term trials because eligible patients are selected from limited potential populations who have rare, severe, or advanced disease. Use of real-world evidence such as from registries and electronic health records will be an important aspect of gene therapy to understand long-term patient outcomes and real-world effectiveness.

## Figures and Tables

**Table 1:** Characteristics, benefits, and challenges of adeno-associated virus-based vectors for gene therapy.

Characteristics	Benefits	Challenges
• Small, non-enveloped, ~4.7-kb single-stranded DNA genome 11, 31 • Genus *Dependoparvovirus* 11 • Belongs to the *Parvoviridae* family 11	• Lack of significant clinical pathogenicity 3 • Predominantly non-integrating (rAAV vector integration into the target cell genome can occur at a rate of 0.1-1% of total transduction) 28, 122 • Ability to package different transgenes 4 • Ability to transduce a wide variety of dividing and non-dividing, terminally differentiated tissues, driving long-term transgene expression 7 • Inefficient at transducing antigen-presenting cells 14 • Exhibits a low immunogenicity profile 3, 5 • Optimized production protocols enable high-titer and high-purity manufacturing 8, 9	• Capsid triggers a dose-dependent immune response 89 • AAV genomes can persist for years in host cells, either episomally or integrated within the host DNA, and be reactivated by a helper virus, such as adenovirus, herpesvirus, human papillomavirus, and vaccinia virus 14, 31 • Due to the broad cross-reactivity between AAV serotypes, NAbs recognizing most serotypes can be found in the majority of subjects 14, 31

AAV, adeno-associated virus; NAb, neutralizing antibody; rAAV, recombinant adeno-associated virus

**Table 2:** AAV therapies approved or in late-stage development

Name	Indication	AAV vector	Company	Status
Voretigene neparvovec	Retinal dystrophy	AAV2	Spark Therapeutics	Approved in Europe 123/Approved in USA 124
Onasemnogene abeparvovec	Spinal muscular atrophy	AAV9	Novartis	Approved in Europe 125/Approved in USA 126
Valoctocogene roxaparvovec	Hemophilia A	AAV5	BioMarin	Approved in Europe 127/ Biologics License Application resubmission in progress in USA 128
Etranacogene dezaparvovec	Hemophilia B	AAV5	UniQure	Conditional approval in Europe 129/Approved in USA 130
Giroctocogene fitelparvovec	Hemophilia A	AAV2/6	Pfizer	Phase 3 clinical: NCT03861273 131
Fidanacogene elaparvovec	Hemophilia B	AAVRh74var (also known as AAV-Spark100)	Pfizer	Phase 3 clinical: NCT03861273 132
Lenadogene nolparvovec	Leber's congenital amaurosis type 2	rAAV2/2-ND4	GenSight Biologics	Phase 3 clinical: NCT02652767, 133 NCT02652780, 134 NCT03406104, 135 NCT03293524 136
Delandistrogene moxeparvovec	Duchenne muscular dystrophy	AAVRh74	Serepta Therapeutics	Approved in USA 137
Eladocagene exuparvovec	Aromatic L-amino acid decarboxylase (AADC) deficiency	AAV2	PTC Therapeutics	Approved in Europe 138

AAV, adeno-associated virus; rAAV2, recombinant adeno-associated virus 2.

**Table 3:**
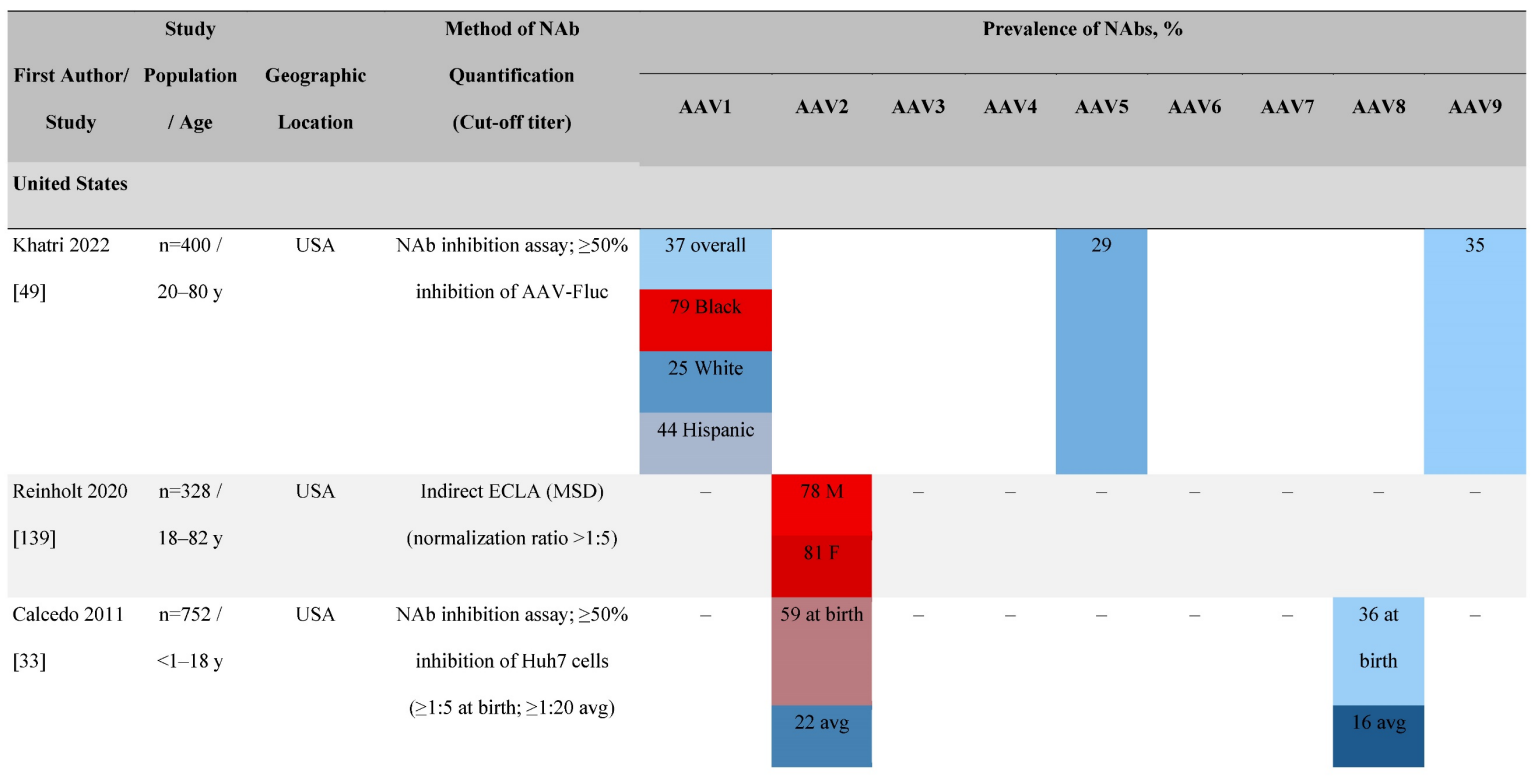

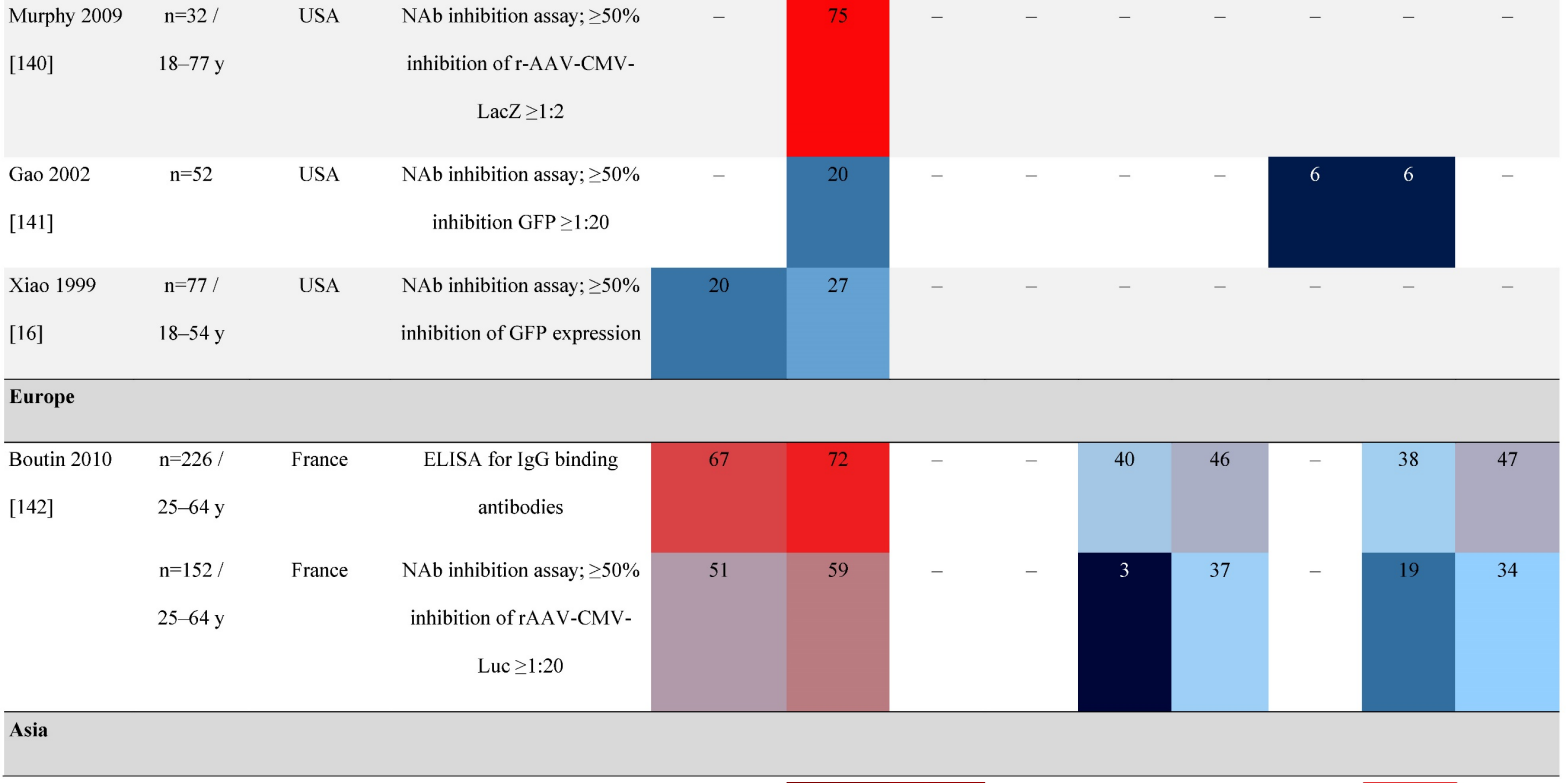

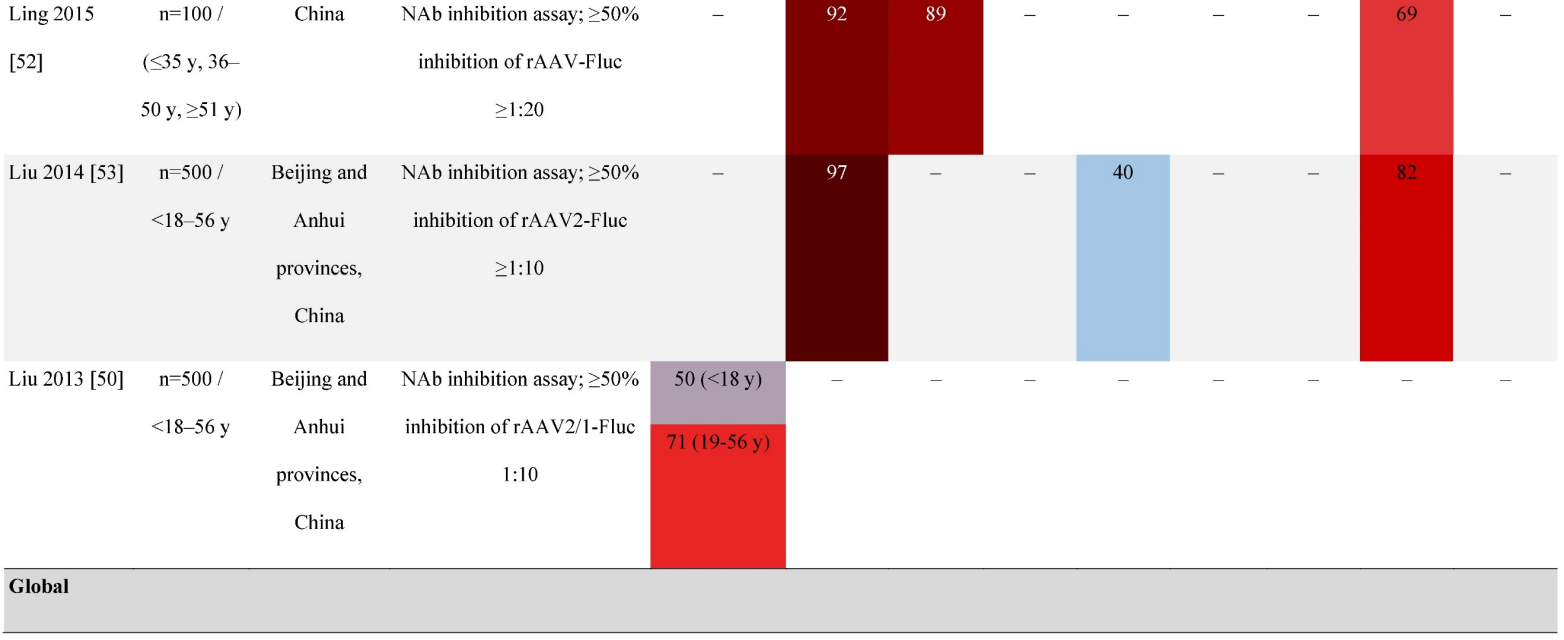

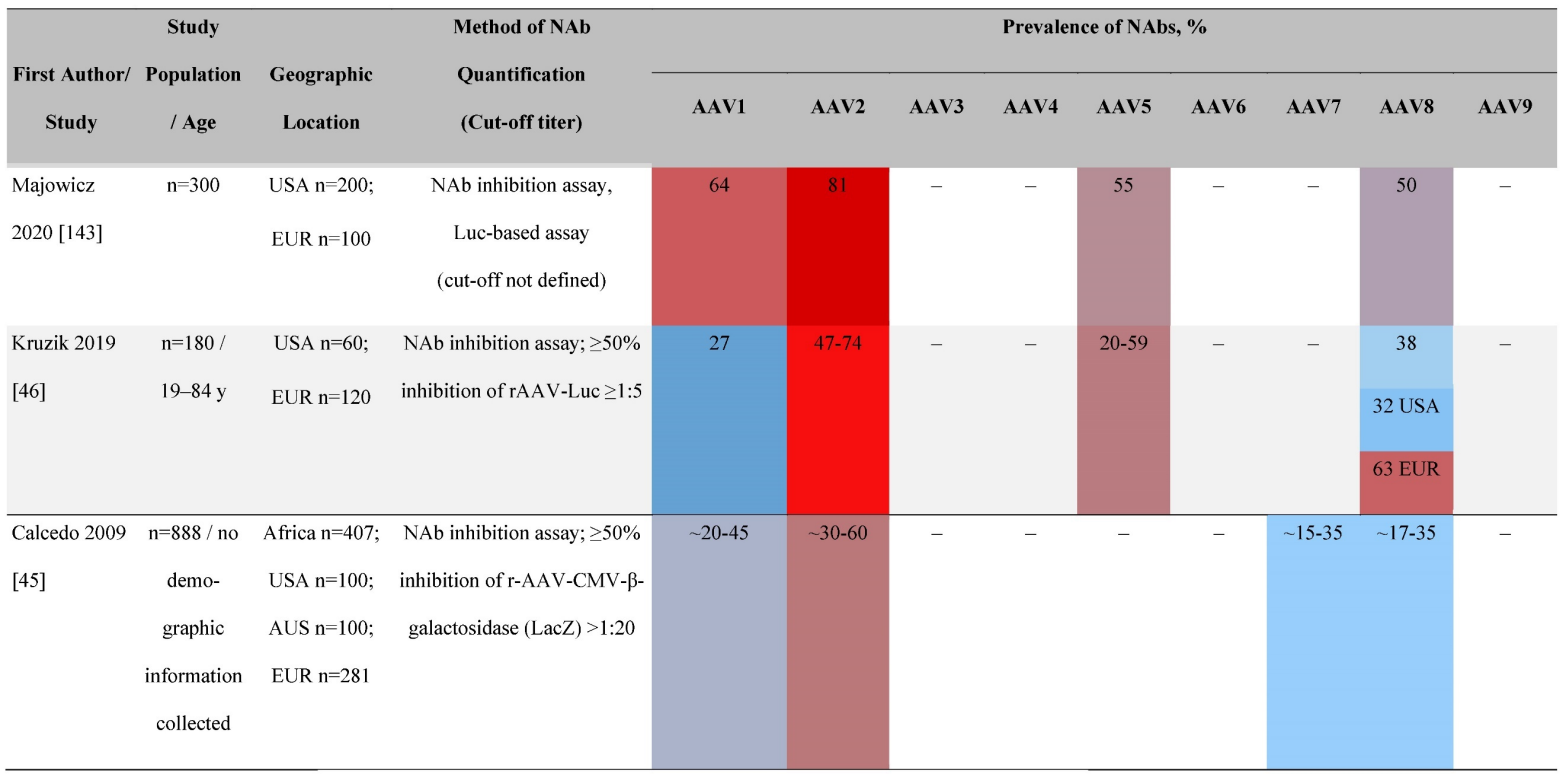
Seroprevalence of NAbs against AAVs in healthy subjects

**Prevalence color coding:** 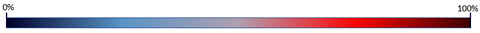
AAV, adeno-associated virus; AUS, Australia; avg, average across all ages; CMV, cytomegalovirus; ECLA, electrochemiluminescence assay; ELISA, enzyme-linked immunosorbent assay; EUR, Europe; F, female; Fluc, firefly luciferase; GFP, green fluorescent protein; IgG, immunoglobulin G; LacZ, β-galactosidase; Luc, luciferase; M, male; MSD, Meso Scale Discovery; NAb, neutralizing antibody; rAAV, recombinant adeno-associated virus; S Africa, South Africa.

**Table 4:**
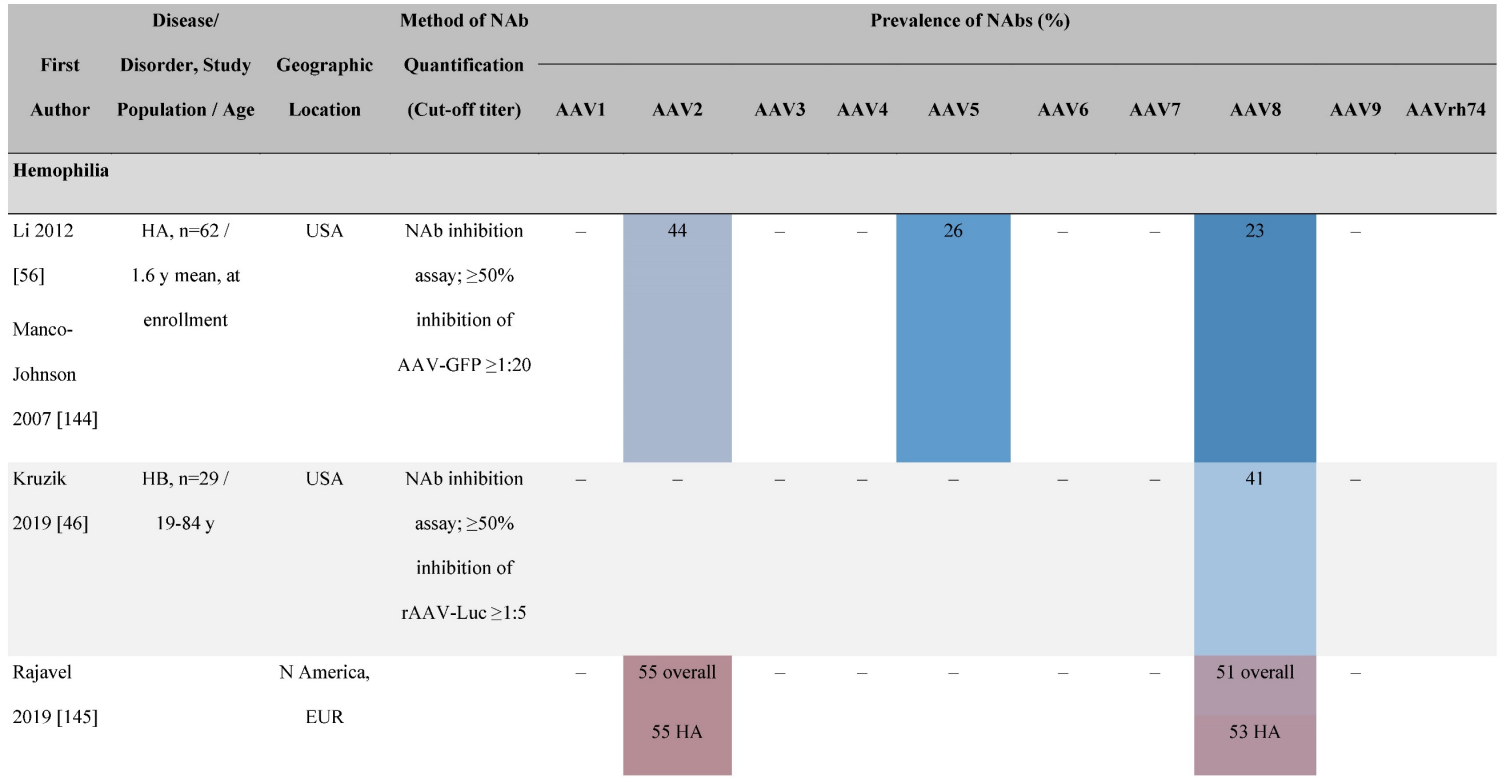

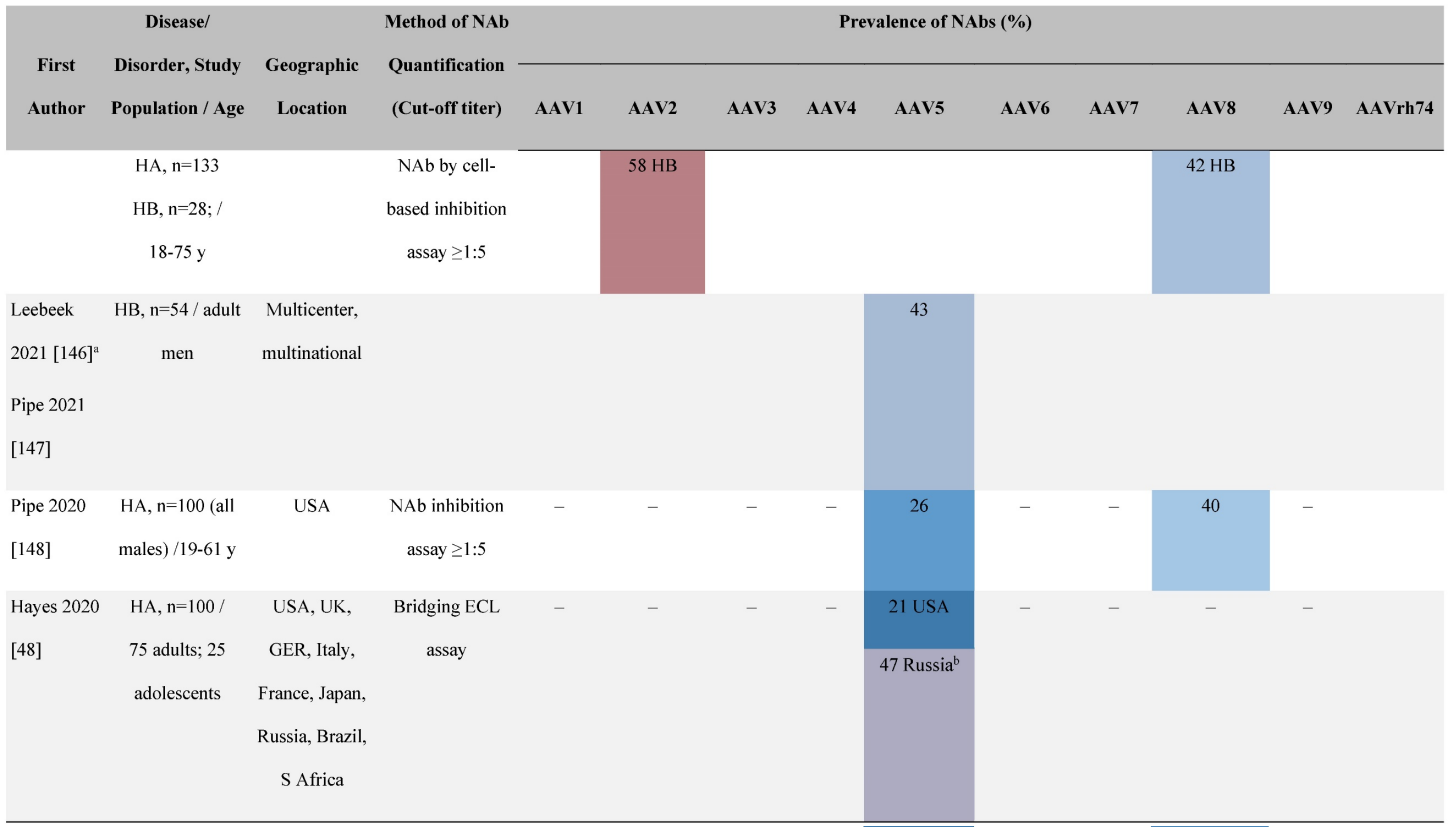

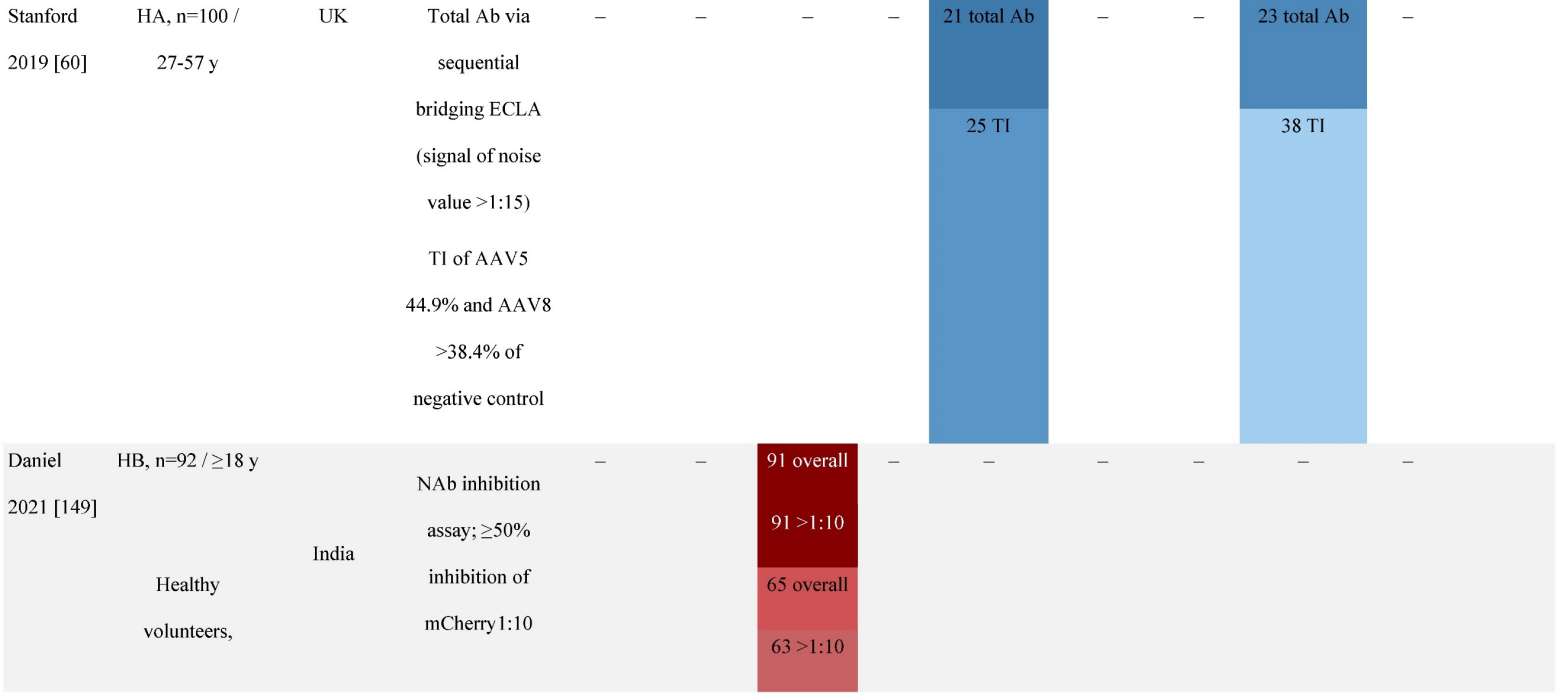

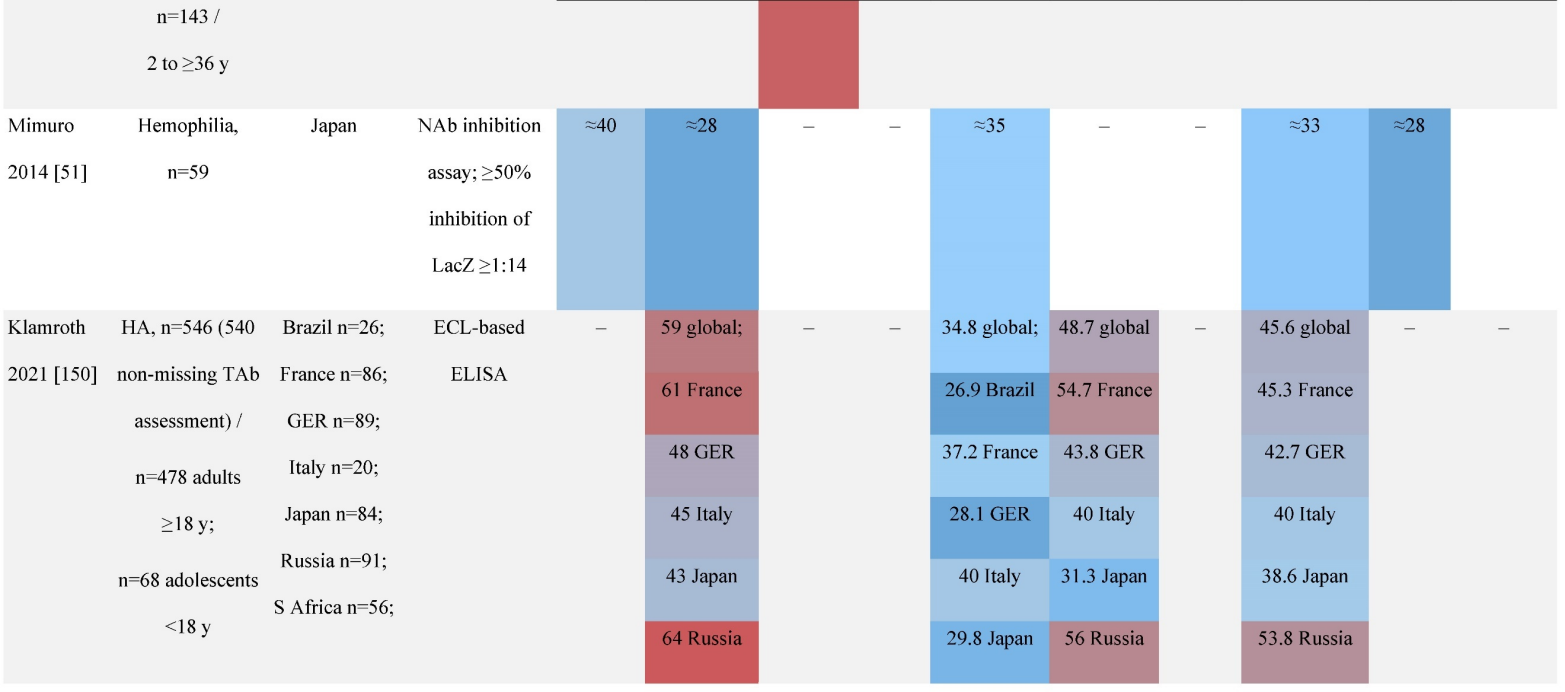

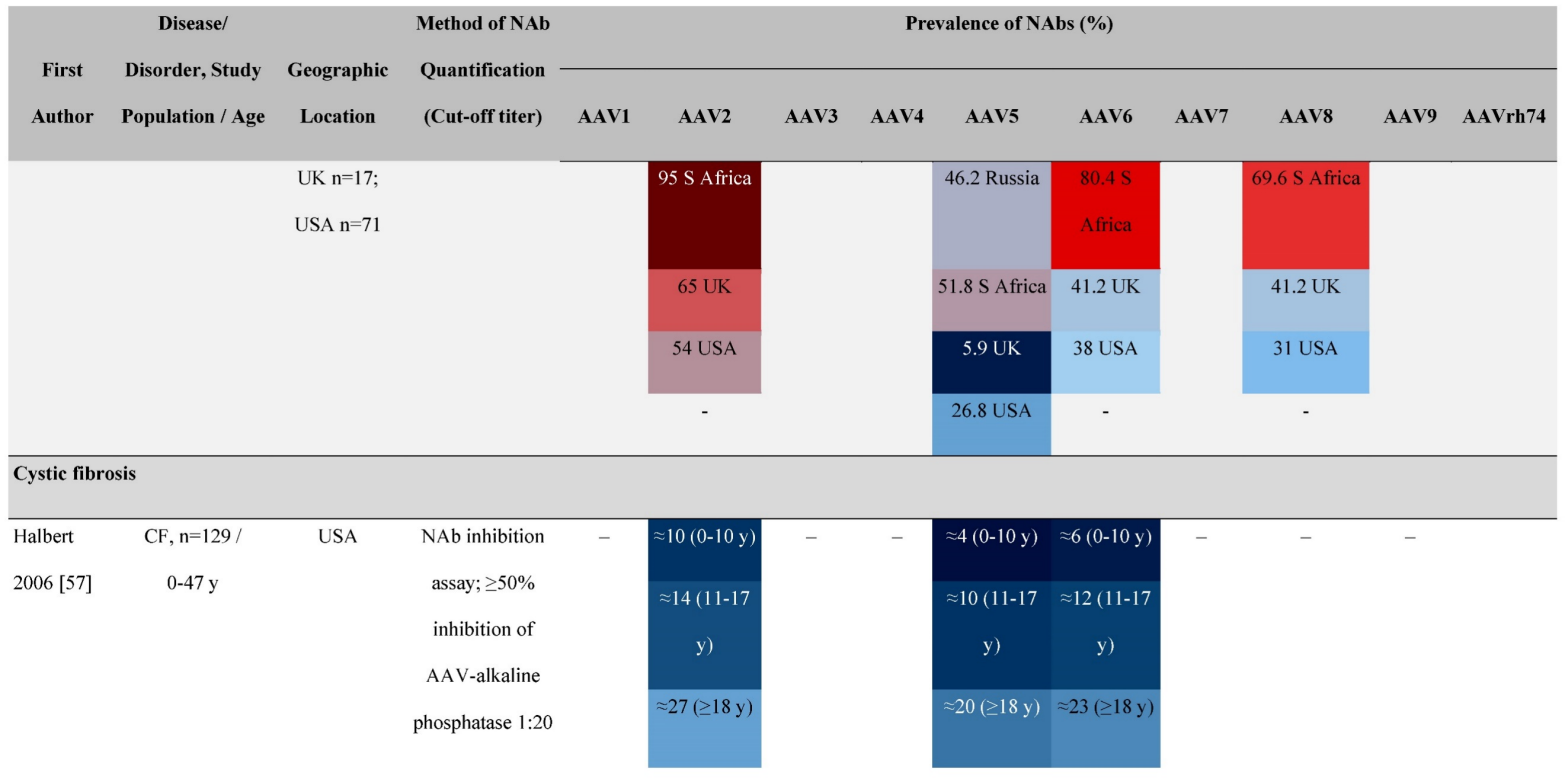

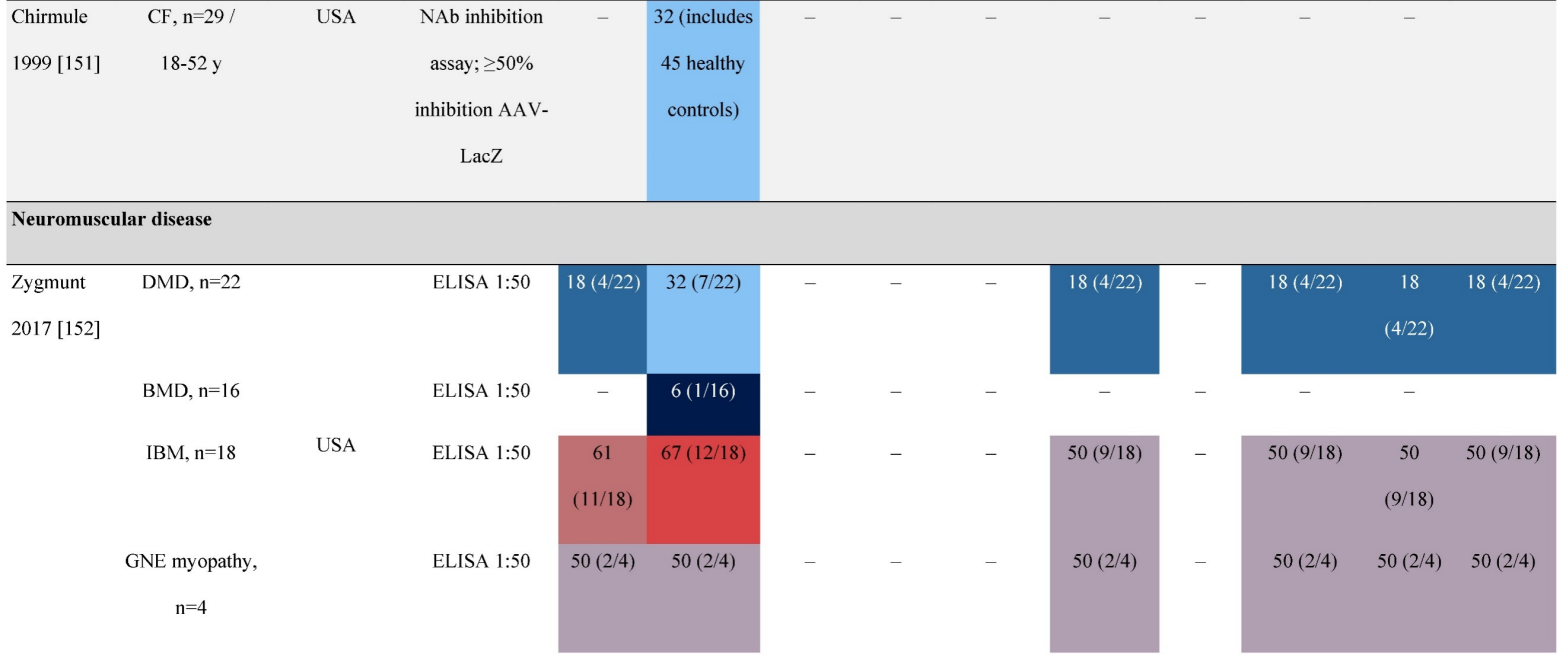

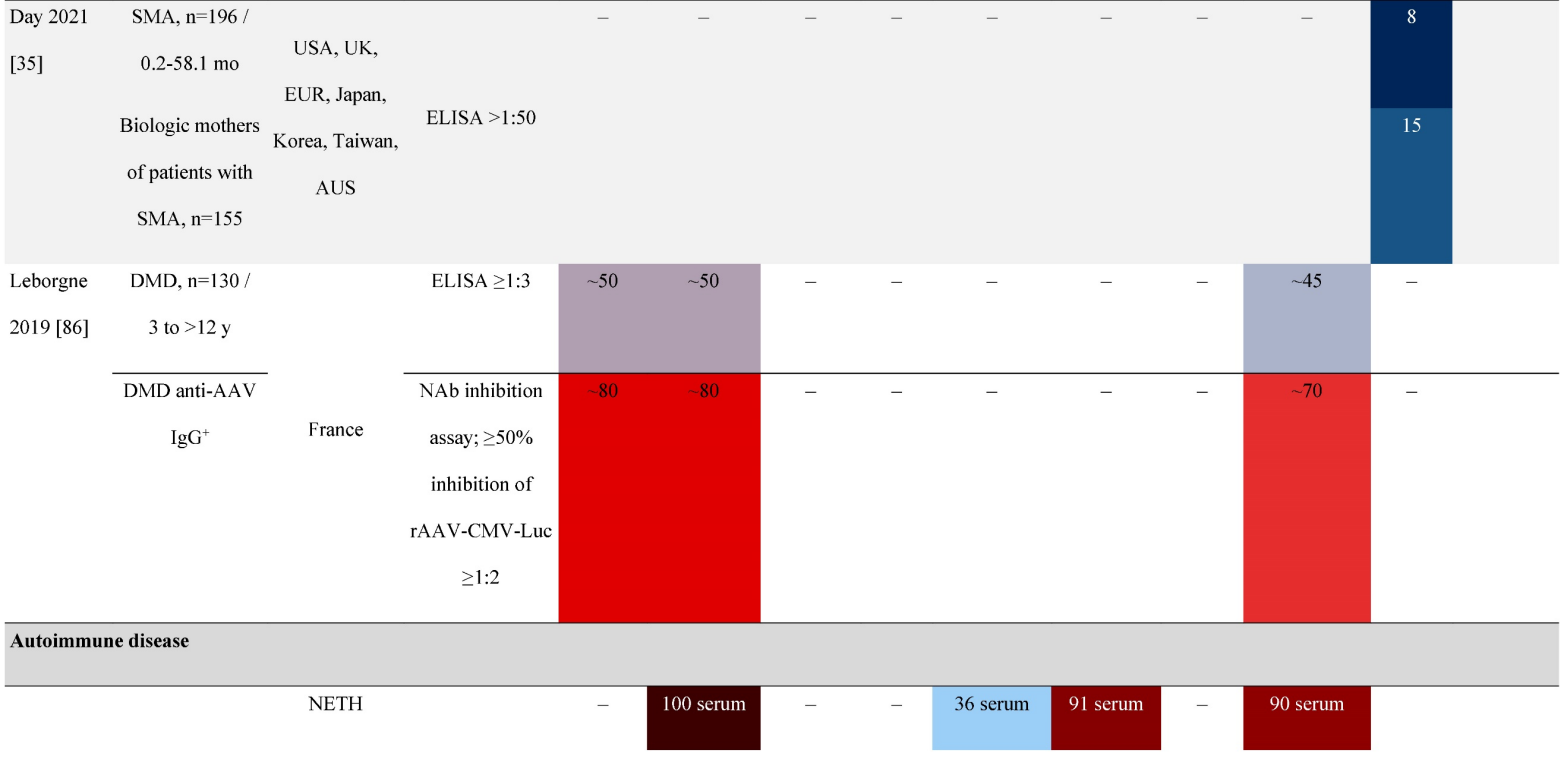

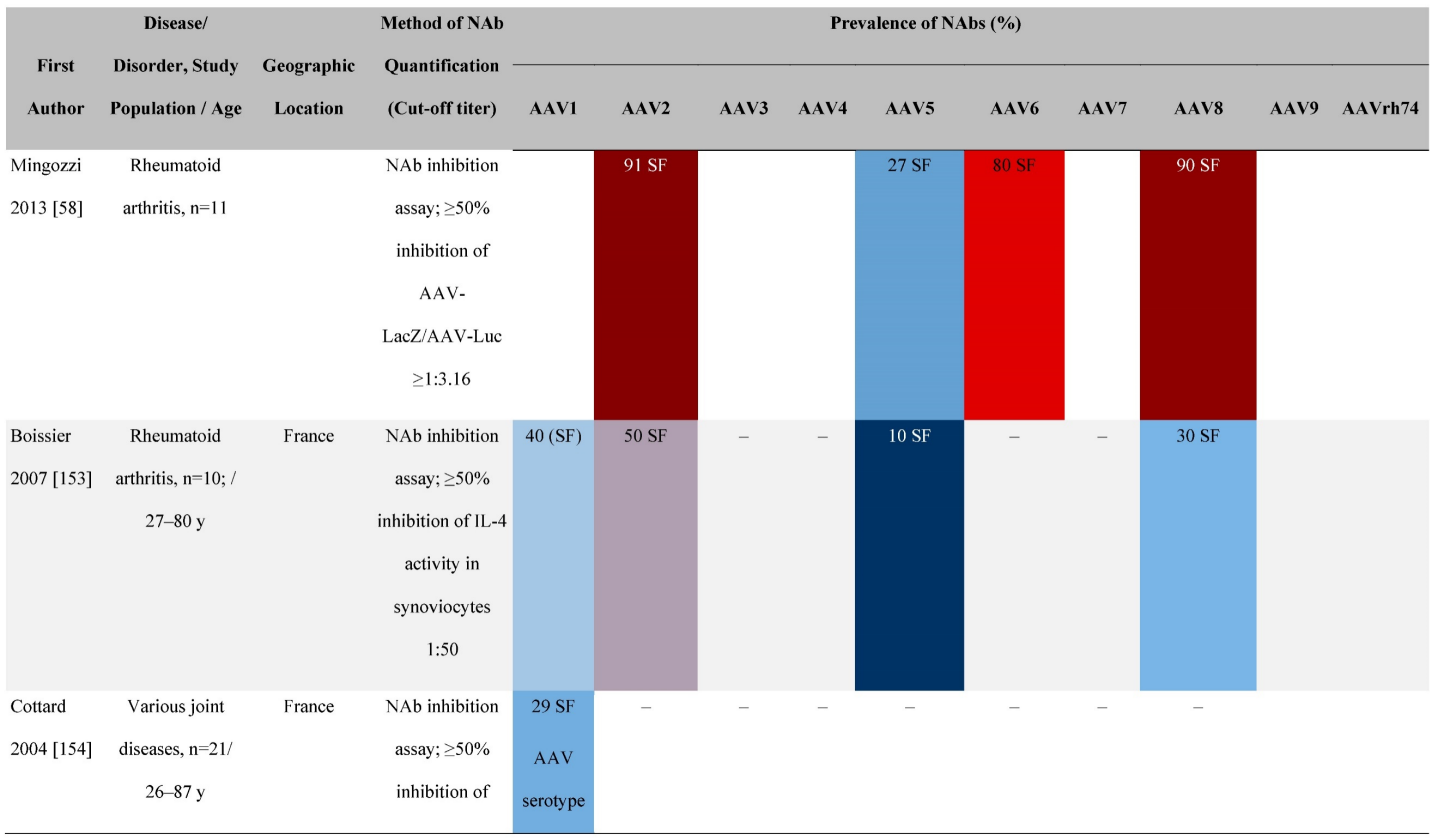

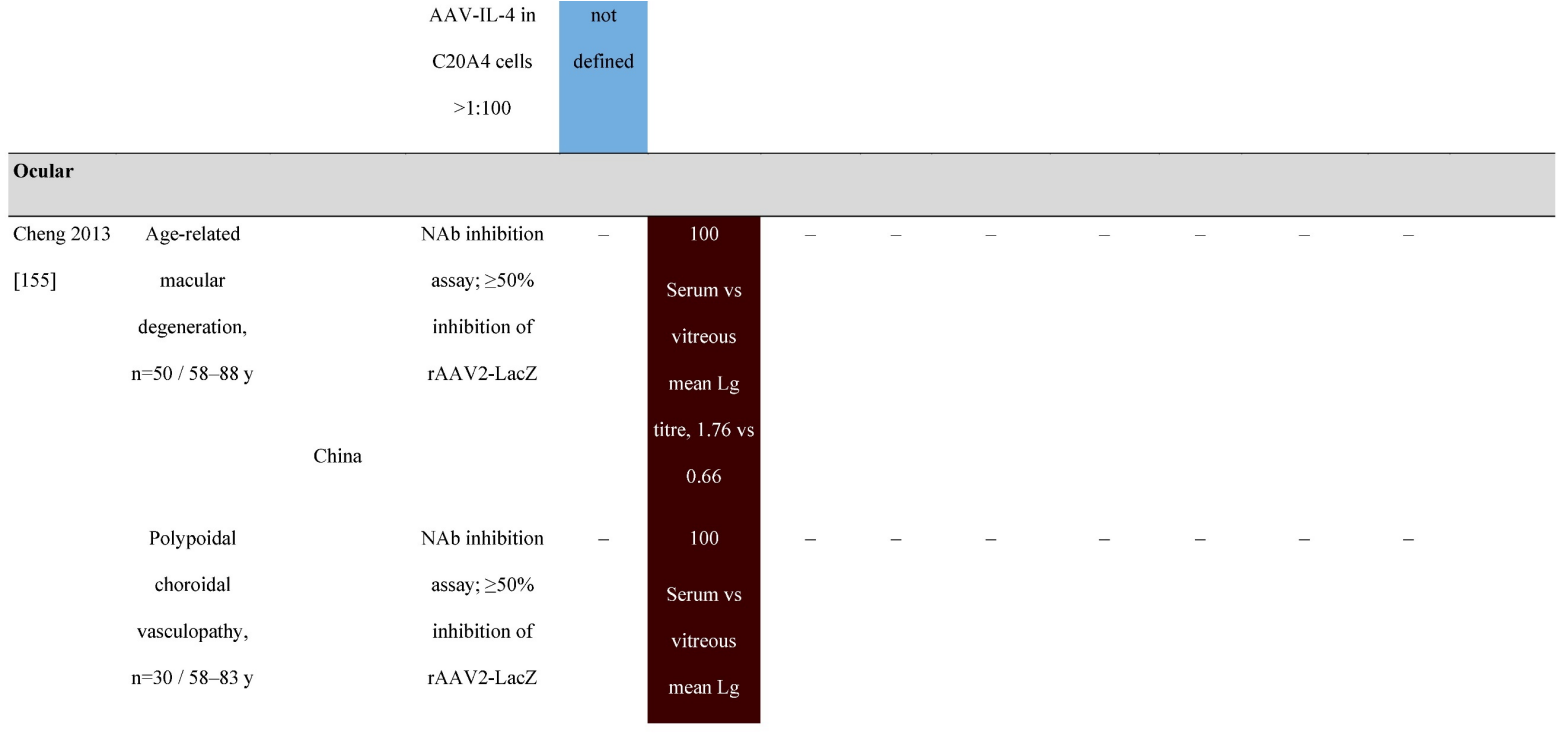

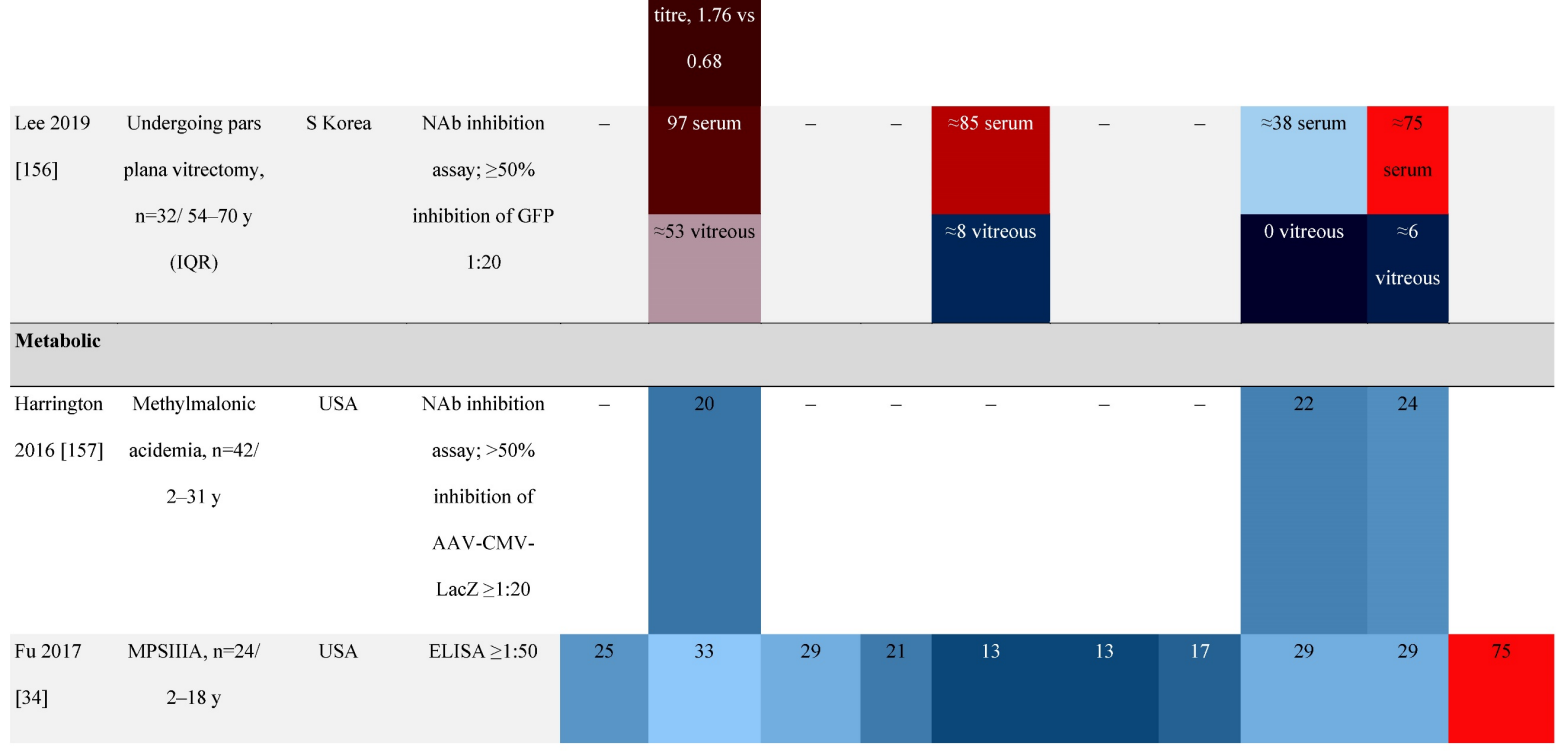

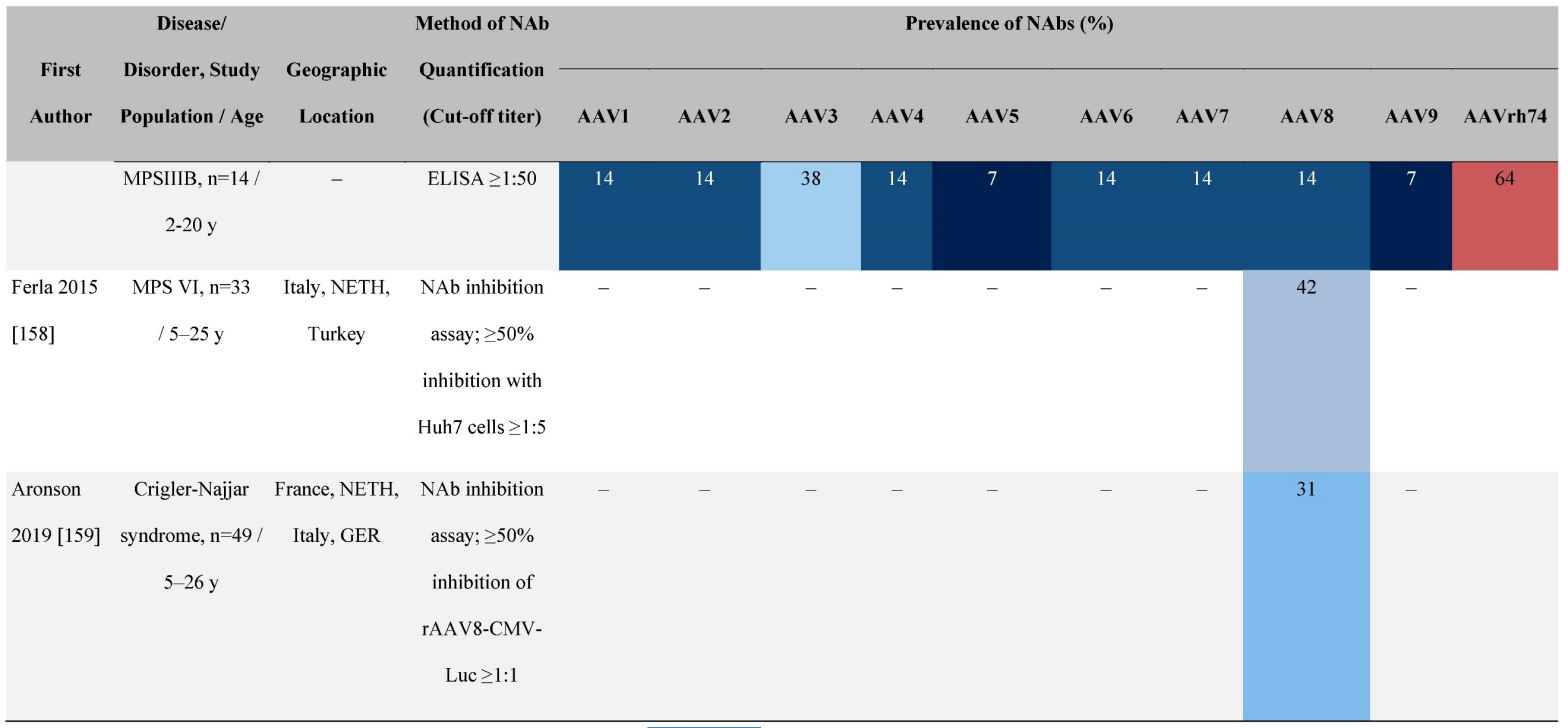

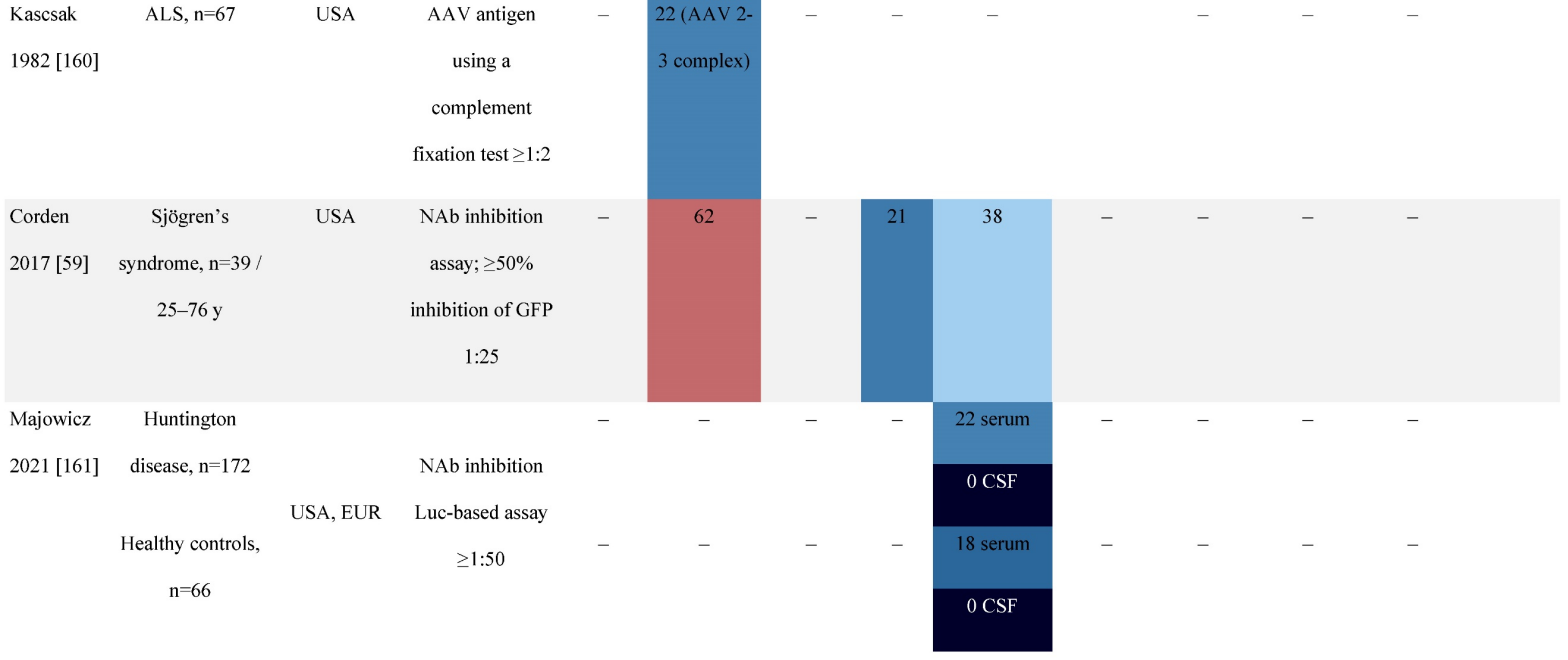

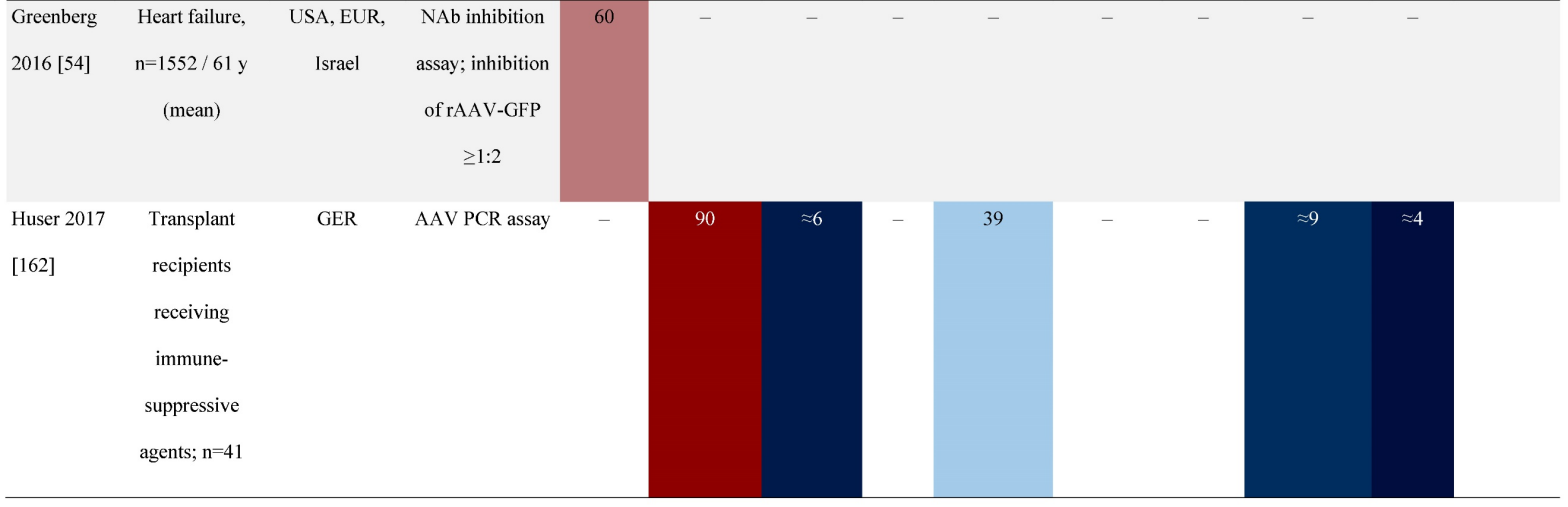

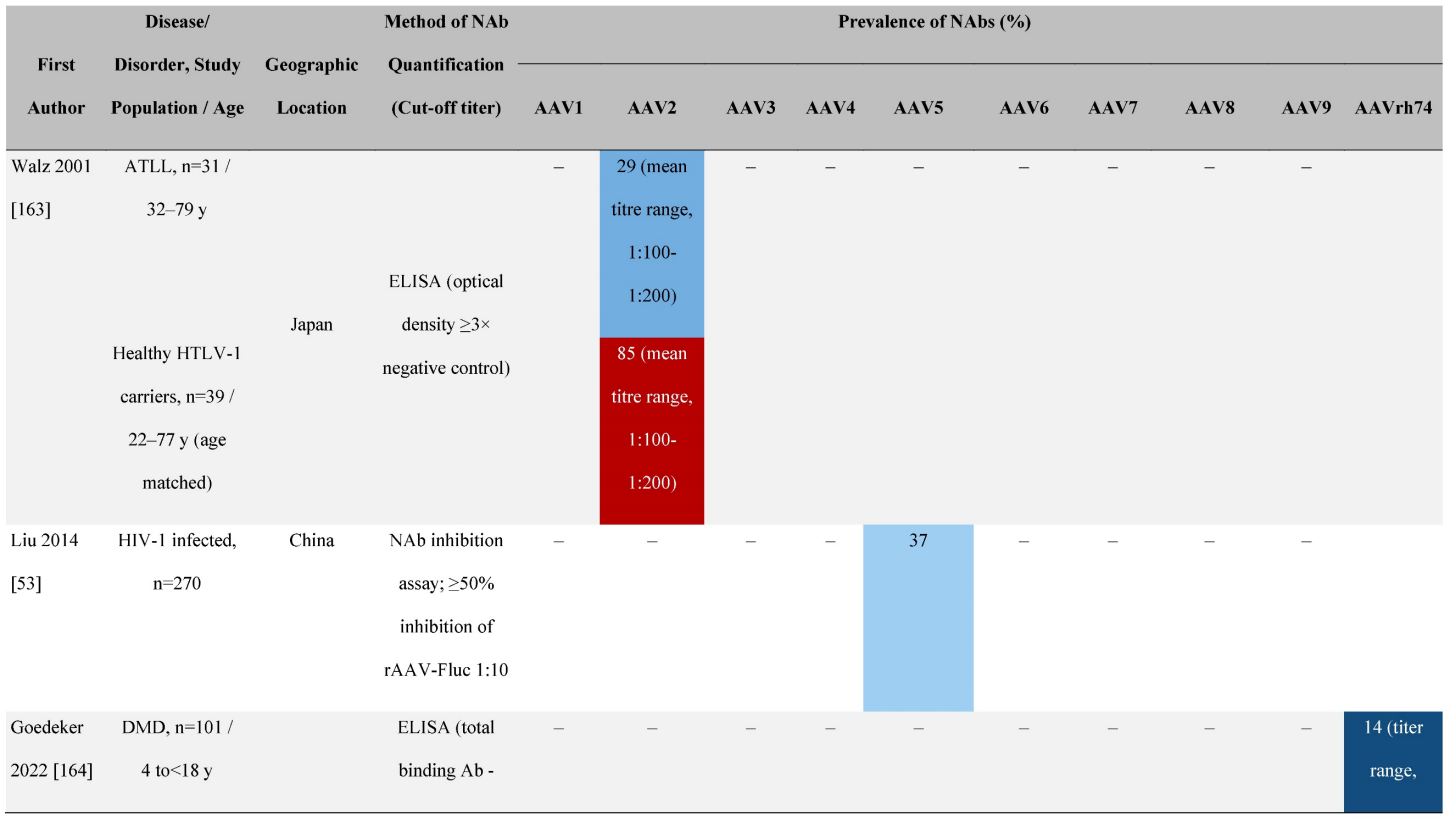

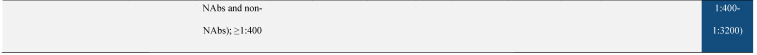
Seroprevalence of NAbs against AAVs in patients with a monogenic disorder

**Prevalence color coding:** 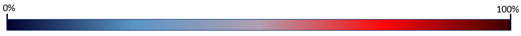
^a^ Method of NAb quantification not reported. ^b^ Data reported only for United States (lowest) and Russian Federation (highest). AAV, adeno-associated virus; Ab, antibody; ALS, amyotrophic lateral sclerosis; ATLL, adult T-cell leukemia lymphoma; AUS, Australia; BMD, Becker muscular dystrophy; CF, cystic fibrosis; CMV, cytomegalovirus; CSF, cerebrospinal fluid; DMD, Duchenne muscular dystrophy; ECL, electrochemiluminescent; ECLA, electrochemiluminescence assay; ELISA, enzyme-linked immunosorbent assay; EUR, Europe; F, female; Fluc, firefly luciferase; GER, Germany; GFP, green fluorescent protein; GNE, UDP-*N*-acetylglucosamine 2-epimerase/*N*-acetylmannosamine kinase; HA, hemophilia A; HB, hemophilia B; HIV, human immunodeficiency virus; HTLV, human T cell lymphotropic virus; IBM, inclusion body myositis; IgG, immunoglobulin G; IL, interleukin; IQR, interquartile range; LacZ, β-galactosidase; Lg, log10; Luc, luciferase; M, male; MPS, mucopolysaccharidosis; NAb, neutralizing antibody; NETH, Netherlands; rAAV, recombinant adeno-associated virus; S Africa, South Africa; SF, synovial fluid; SMA, spinal muscular atrophy; TAb, total antibody; TI, transduction inhibition.

**Table 5:**
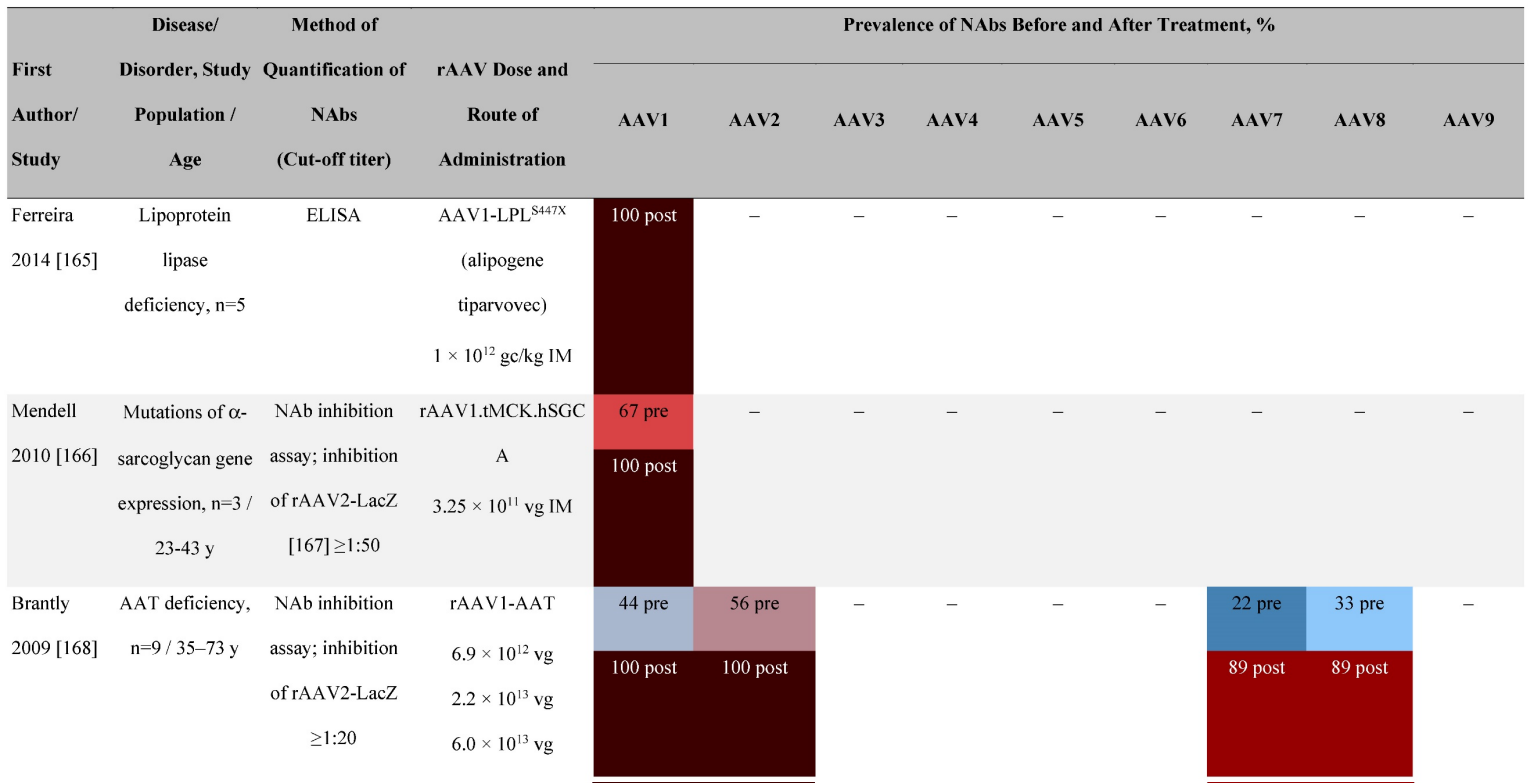

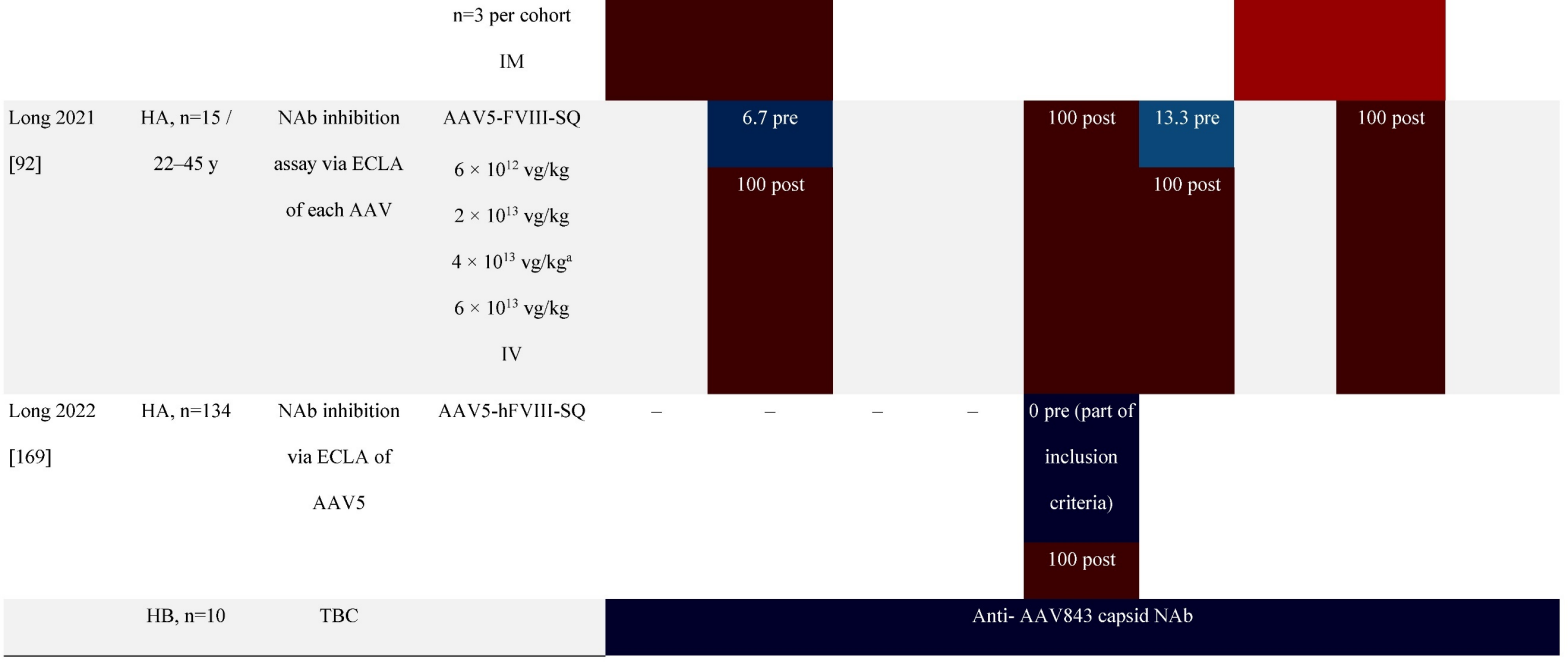

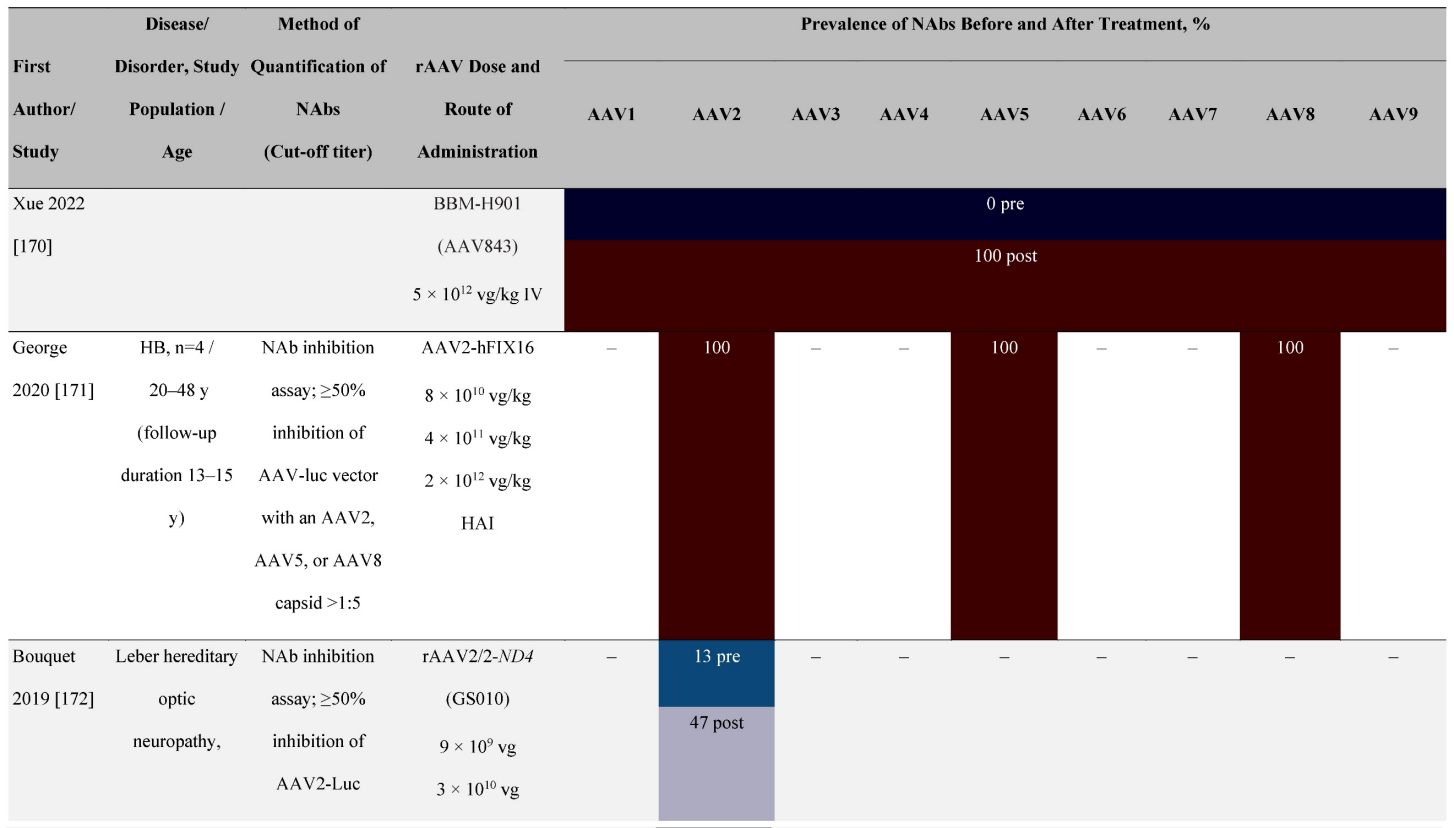

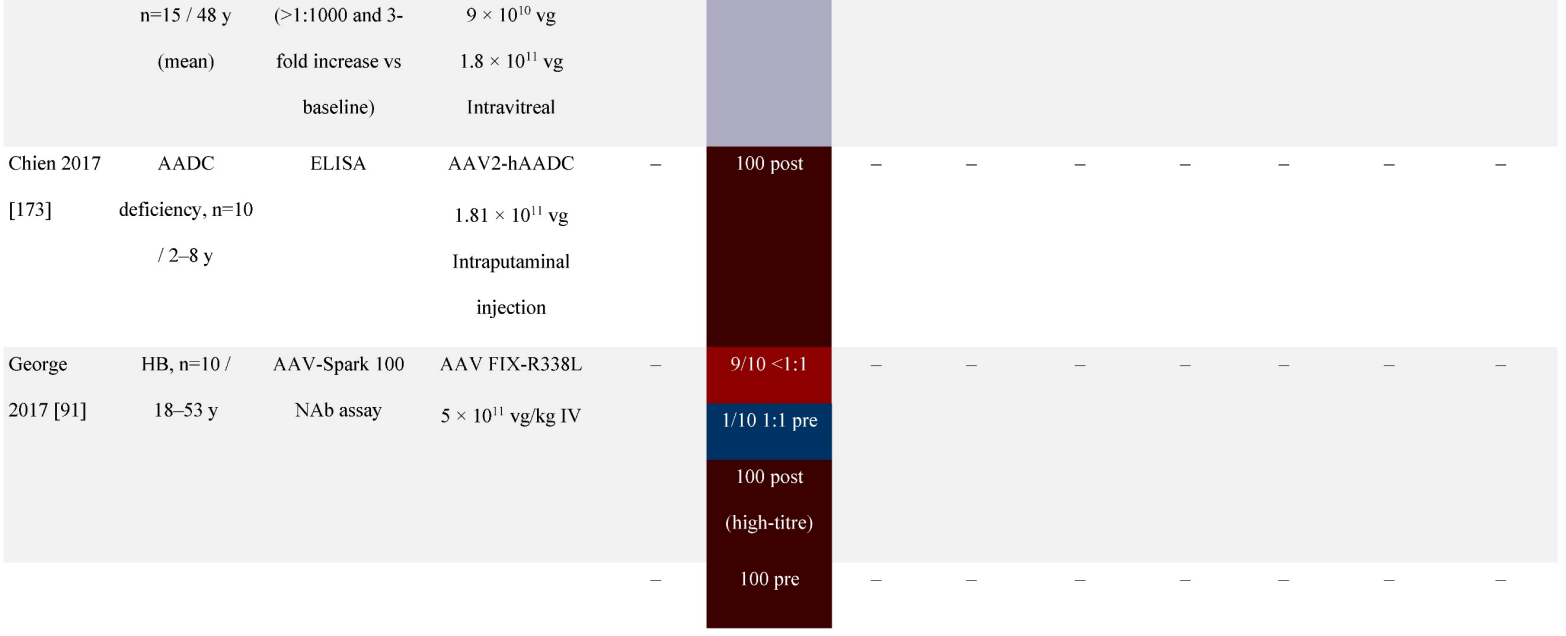

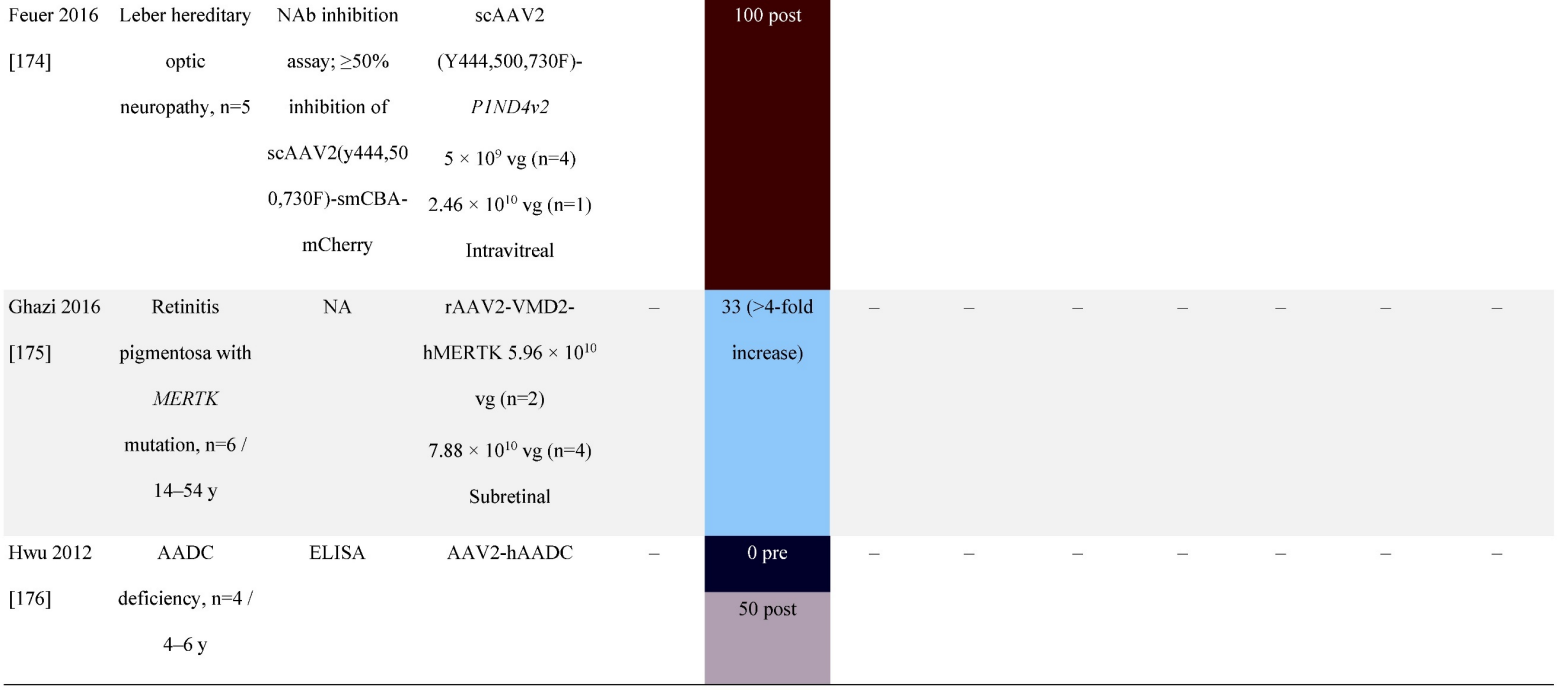

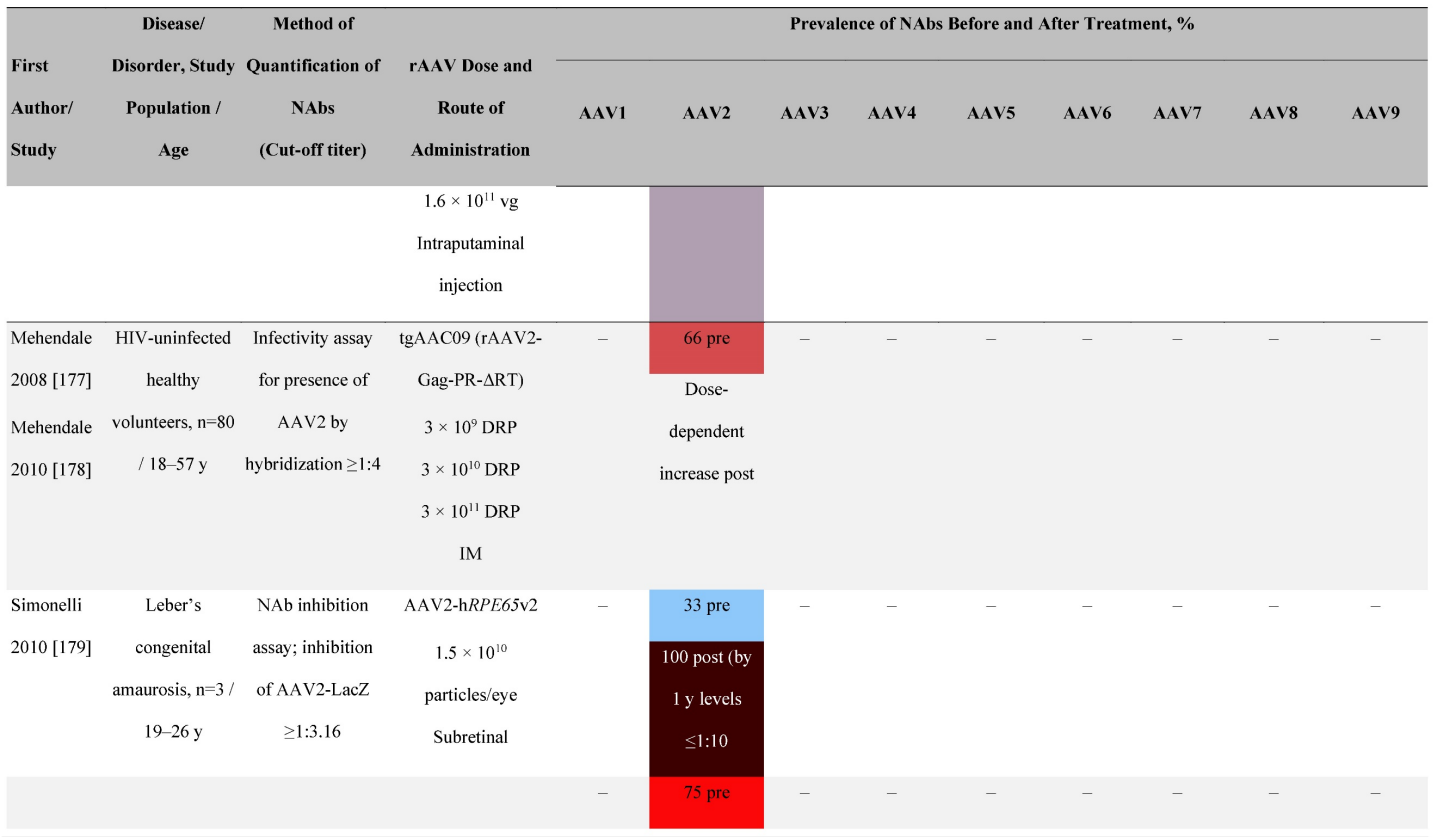

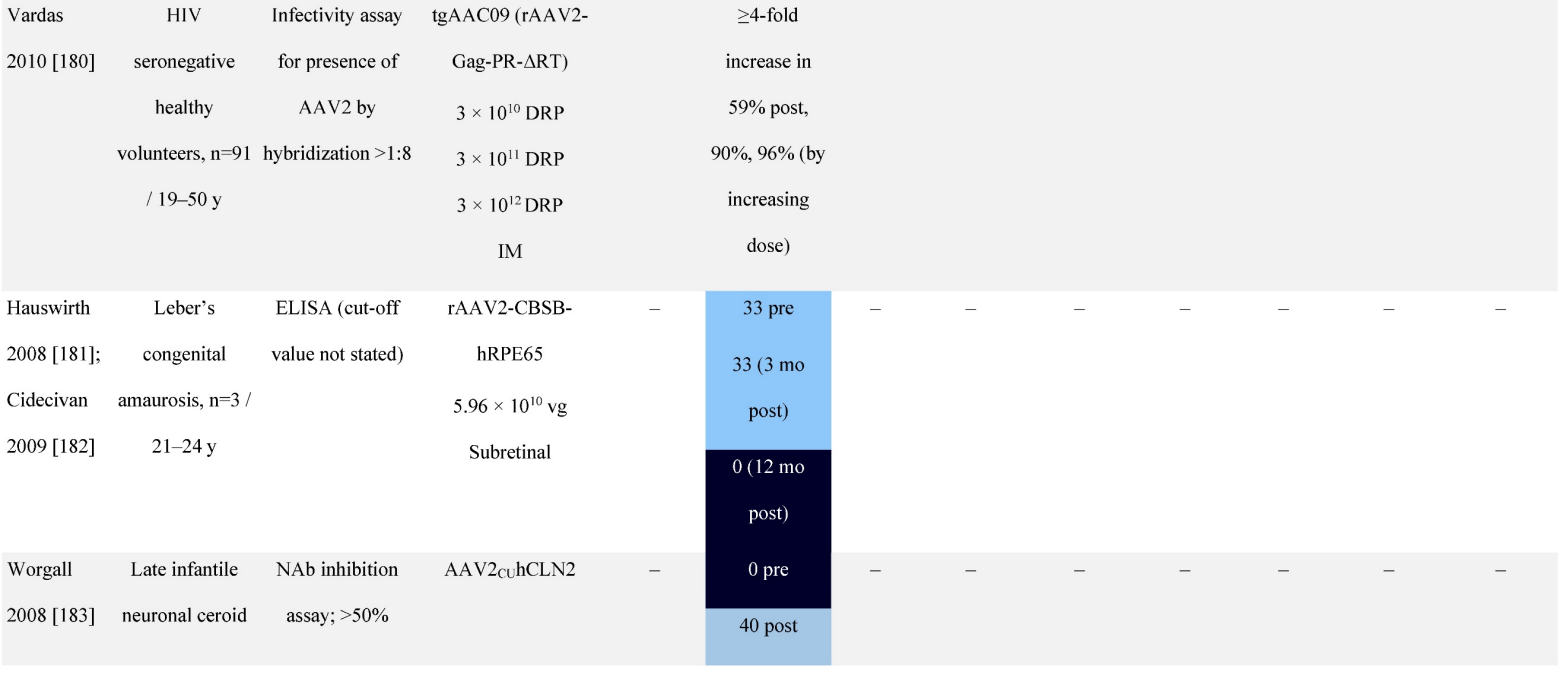

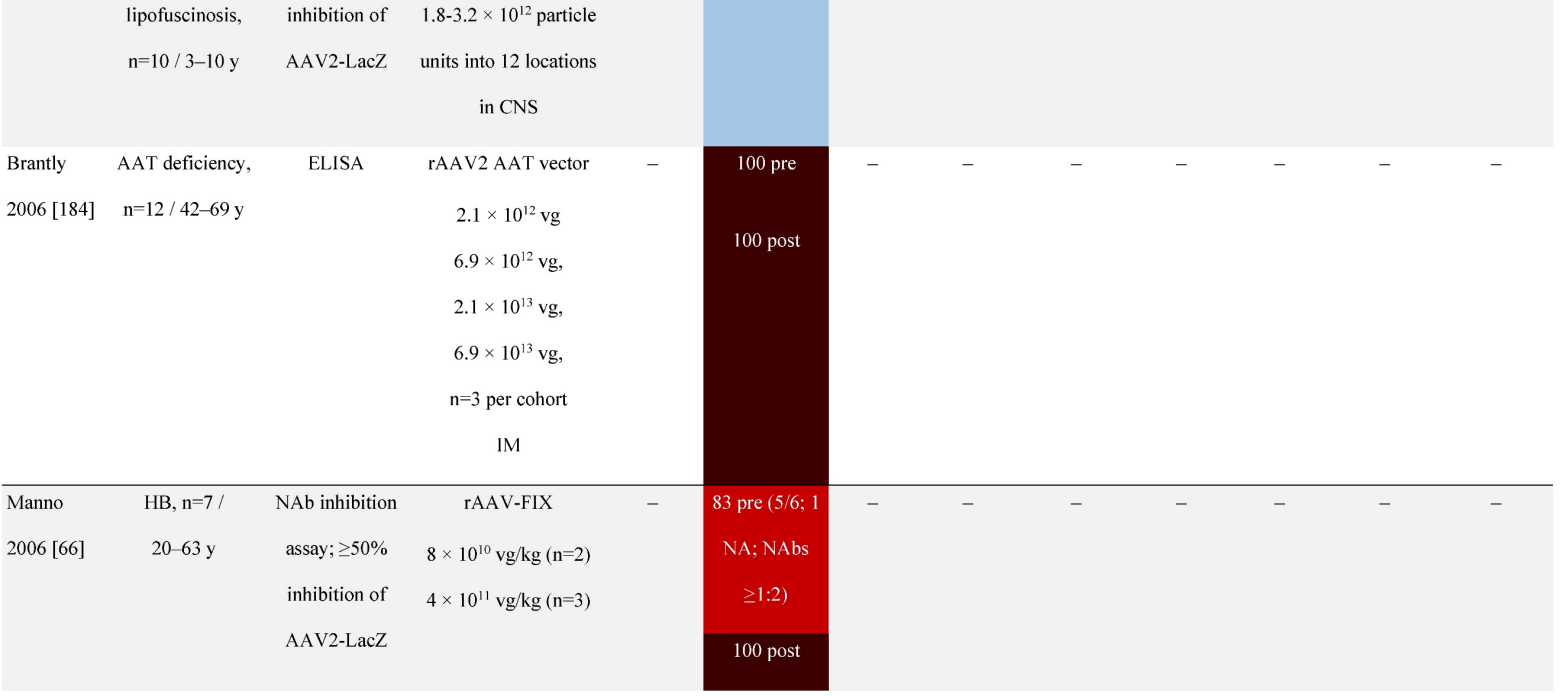

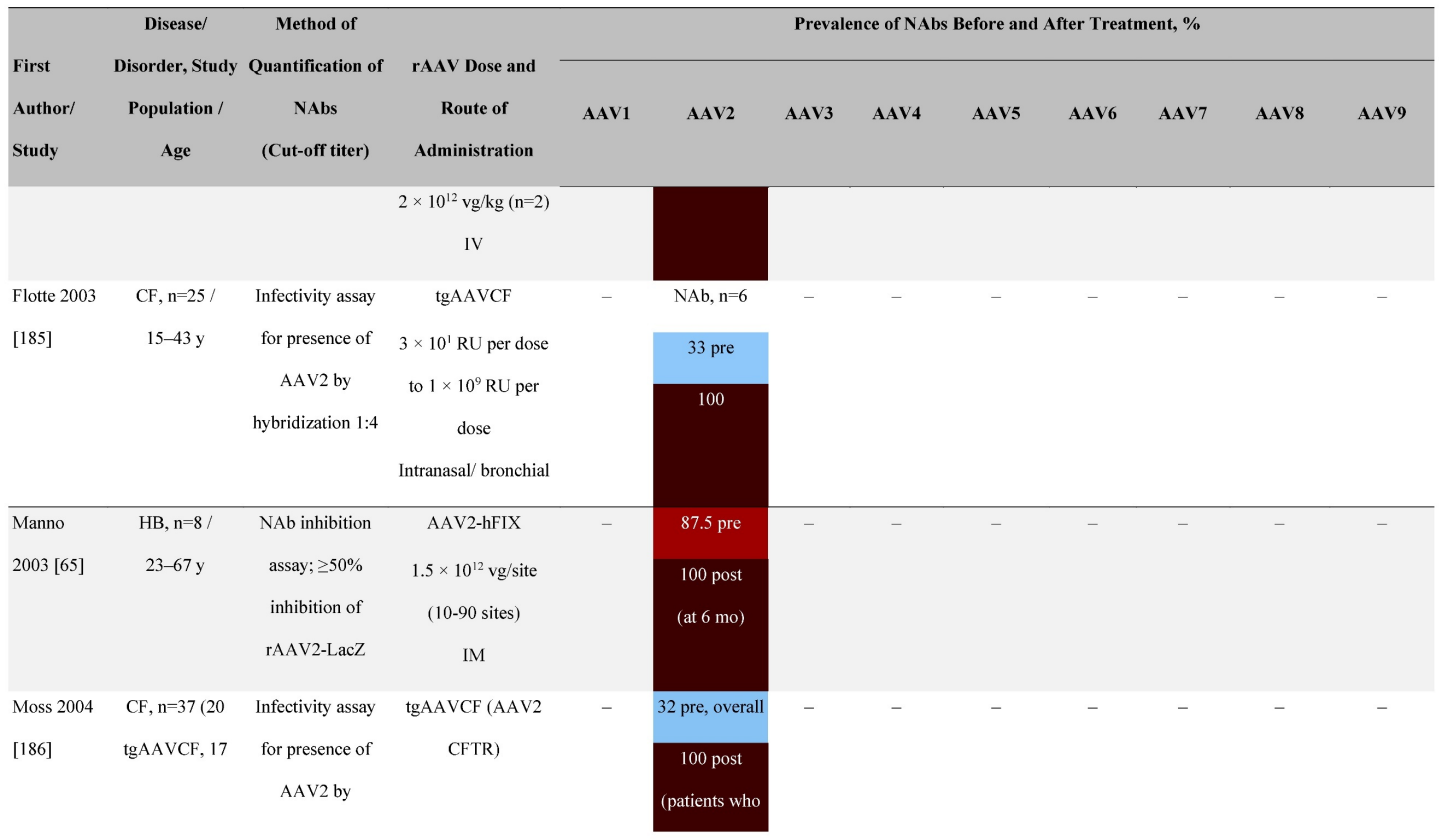

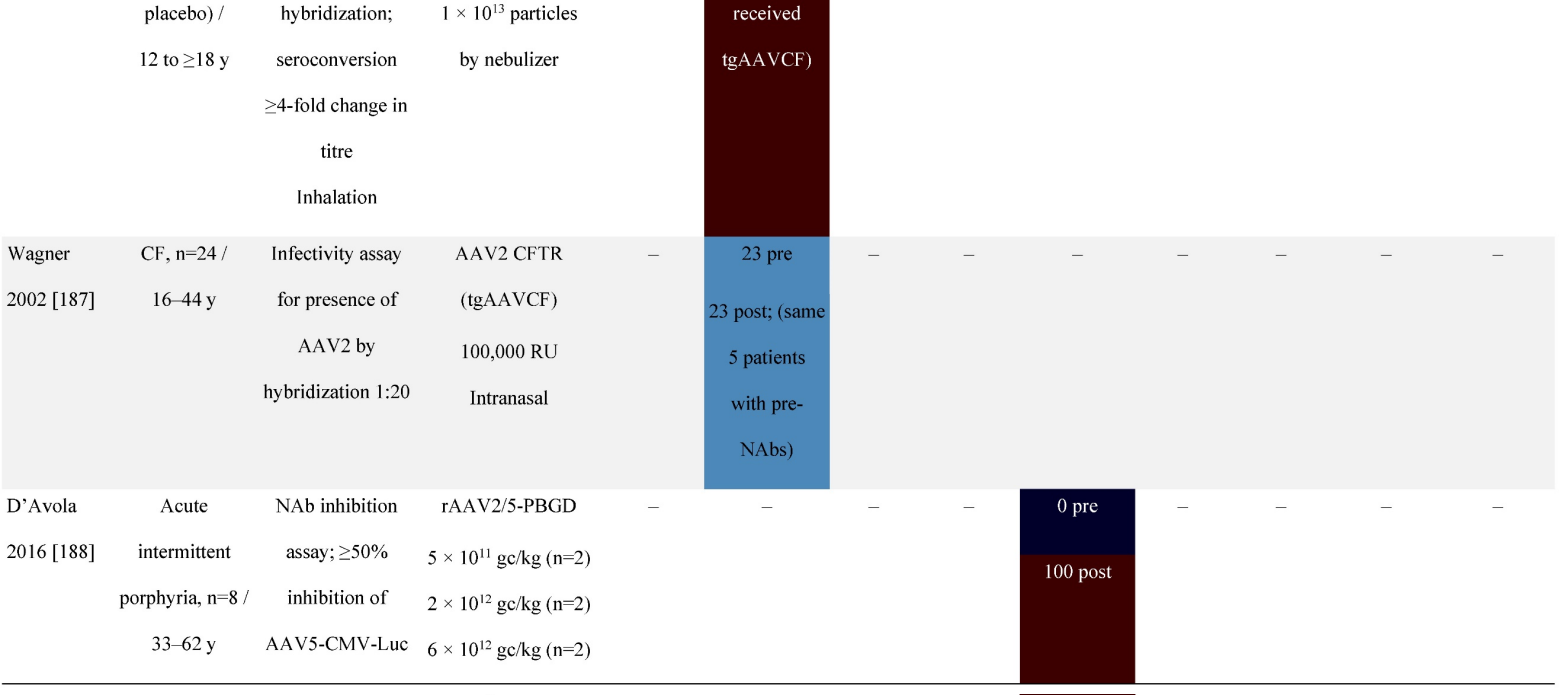

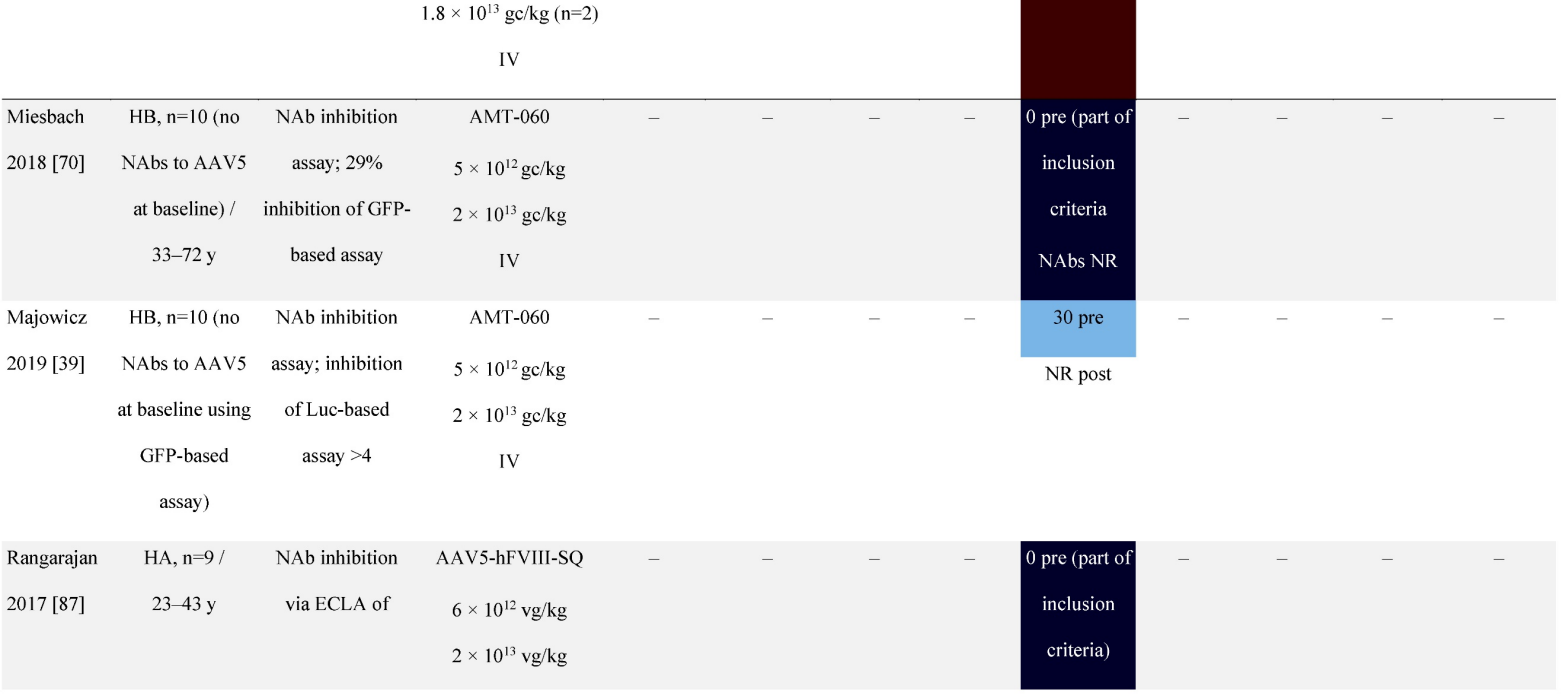

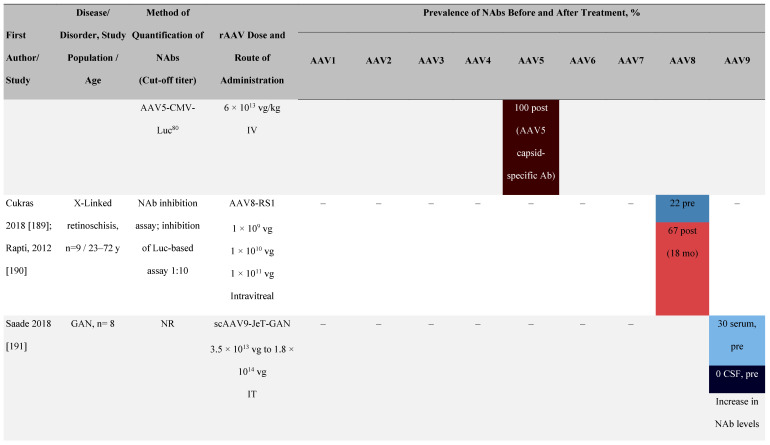

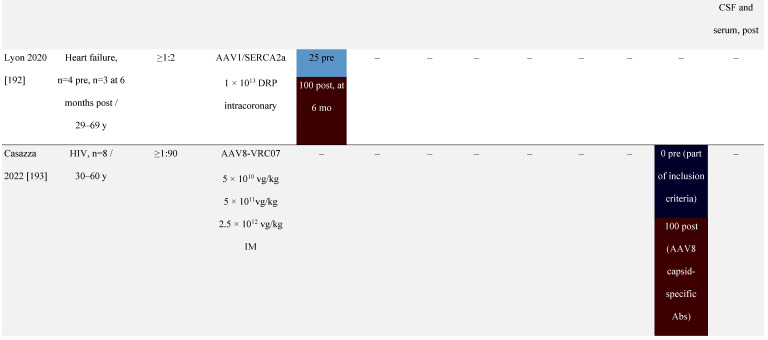

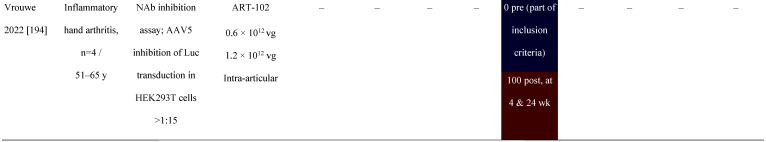
Seroprevalence of NAbs against AAVs following administration of recombinant therapeutic AAV vector serotypes

**Prevalence color coding:** 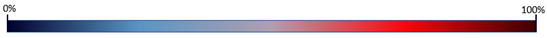
^a^ ECLA data were not available for 2 patients in this cohort. AADC, aromatic L-amino acid decarboxylase; AAT, α-1 antitrypsin; AAV, adeno-associated virus; Ab, antibody; CF, cystic fibrosis; CFTR, cystic fibrosis transmembrane conductance regulator; CMV, cytomegalovirus; CNS, central nervous system; CSF, cerebrospinal fluid; DRP, DNase-resistant particles; ECLA, electrochemiluminescence assay; ELISA, enzyme-linked immunosorbent assay; GAN, giant axonal neuropathy; HA, hemophilia A; HB, hemophilia B; HIV, human immunodeficiency virus; gc, genome copies; GFP, green fluorescent protein; HAI, hepatic artery infusion; IM, intramuscular; IT, intrathecal; IV, intravenous; LacZ, β-galactosidase; Luc, luciferase; NA, not available; NAb, neutralizing antibody; NR, not reported; PBGD, porphobilinogen deaminase; rAAV, recombinant adeno-associated virus; RU, replication units; SQ, subcutaneous; vg, vector genomes.

**Table 6:** Strategies under investigation to mitigate pre-existing anti-AAV NAbs and humoral immunity to rAAV vectors post-gene therapy 5, 77, 79, 113, 114, 121

	Strategy	Considerations
**Strategies to mitigate pre-existing anti-AAV NAbs**	Route of administration	• Easy to implement in clinical practice • May alter the pattern of transduction, limiting gene delivery to target cell type or tissue • Limited number of routes of administration depending on the target tissue • Target 'immune privileged' tissues
	Alternative AAV vectors	• Increasing number of novel AAV vectors identified which may be more effective at evading NAbs. • Increasing understanding of AAV epitopes is enabling rational mutation of antigenic regions - could be applied to any vector. • In vitro and in vivo screens available to identify NAb-resistant vectors • Engineering novel AAVs can be expensive and technically challenging; novel AAVs may have unwanted/unintended properties e.g., toxicity issues
	AAV modification	• Non-genetic/chemical modifications are amenable to scale-up in manufacturing • Limited resistance of PEGylated AAV to NAbs; examples of other biological polymers are limited to date
	Decoy capsids	• Clinically translatable if 'known' serotype decoy capsids are used • Possible to adjust ratio of decoy to full capsids depending on pre-existing NAb titer • Potential to increase immune responses, including CD8+ T cell activation with destruction of transduced cells and enhanced NAb response • Would require production of more AAV capsids, which could create a manufacturing bottleneck
	Plasmapheresis	• Multiple rounds can reduce pre-existing NAb levels • Relatively non-invasive and safe; routinely used in other applications • Less effective with high pre-existing NAb titers • 'Rebound' phenomenon may limit effectiveness and compromise the option of repeated use
	Broad-acting immunosuppressants	• Approved immunosuppressants can be used • Well-characterized safety and efficacy profiles, with known toxicities • May be ineffective at completely depleting the bone marrow memory B-cells to overcome humoral immunity • Little evidence to support the approach despite efficacy of in pre-immunized models
	Antibody-degrading enzymes	• Effective in preclinical animal studies with low and moderate NAb titer • Clinical evidence of safety in patients undergoing graft rejection • Patients may have pre-existing humoral immunity to, or develop humoral immunity against, the enzyme • May only be partially effective against high titers of NAbs
	Anti-FcRn antibodies	• FcRn1 helps maintain circulating IgG levels; use of anti-FcRn antibodies can potentially reduce NAb titers by ~80% for 60 days • Specific for IgG; does not impact IgA, IgM, IgD, and IgE antibodies
**Strategies to mitigate humoral immunity to rAAV vectors post-gene therapy**	Minimizing vector dosage	• Transgene expression may be achieved with low vector doses + corticosteroids or other mild immunosuppressive regimens • Additional strategies might be required to achieve therapeutic level of transgene expression
	Inhibit B-cell activation	• Inhibiting B-cell function may prevent NAb formation whilst retaining cytotoxic T-cell function • Patients may be vulnerable to opportunistic pathogens and tumorigenesis while B-cell levels recover
	Broad-acting immunosuppressants	• Possible to utilize approved drugs to suppress immune responses; highly translatable. • Well-characterized safety and efficacy profiles and mechanisms of action • Preclinical evidence suggests this approach may not be sufficient to prevent NAb formation or enable vector re-administration. • Outcomes of clinical trials investigating gene delivery to the retina also suggest this approach may not be effective in all patients
	Targeted transgene expression	• Reduce off-target expression of the transgene with gene regulatory elements like tissue- and cell-specific promoters or microRNAs • Targeted gene delivery platforms improve the safety profile • Tissue-specific promoters can be weaker compared to ubiquitous promoters; identification of strong but tissue-specific promoters can be challenging

Adapted from "Humoral immune responses to AAV gene therapy in the ocular compartment" by Whitehead M et al. which is licensed under CC BY 4.0. 5 AAV, adeno-associated virus; FDA, US Food and Drug Administration; MicroRNA, micro ribonucleic acid; NAb, neutralizing antibody; PEG, polyethylene glycol.

## Abbreviations

AAP: assembly-activating protein
AAV: adeno-associated virus
APC: antigen-presenting cells
ELISA: enzyme-linked immunosorbent assay
G3BP: galectin 3 binding protein
GFP: green fluorescent protein
IC50: half maximal inhibitory concentration
ITR: inverted terminal repeats
MAAP: membrane-associated accessory protein
MHC: Major histocompatibility complex
MOI: multiplicity of infection
Nab: neutralizing antibody
rAAV: recombinant adeno-associated virus
Rep: replica proteins
ssDNA: single-stranded DNA
TLR: toll-like receptor
